# Endophytic Fungal Communities Associated with Vascular Plants in the High Arctic Zone Are Highly Diverse and Host-Plant Specific

**DOI:** 10.1371/journal.pone.0130051

**Published:** 2015-06-12

**Authors:** Tao Zhang, Yi-Feng Yao

**Affiliations:** 1 State Key Laboratory of Mycology, Institute of Microbiology, Chinese Academy of Sciences, Beijing, China; 2 Institute of Medicinal Biotechnology, Chinese Academy of Medical Sciences & Peking Union Medical College, Beijing, China; 3 State Key Laboratory of Systematic and Evolutionary Botany, Institute of Botany, Chinese Academy of Sciences, Beijing, China; Graz University of Technology (TU Graz), AUSTRIA

## Abstract

This study assessed the diversity and distribution of endophytic fungal communities associated with the leaves and stems of four vascular plant species in the High Arctic using 454 pyrosequencing with fungal-specific primers targeting the ITS region. Endophytic fungal communities showed high diversity. The 76,691 sequences obtained belonged to 250 operational taxonomic units (OTUs). Of these OTUs, 190 belonged to Ascomycota, 50 to Basidiomycota, 1 to Chytridiomycota, and 9 to unknown fungi. The dominant orders were Helotiales, Pleosporales, Capnodiales, and Tremellales, whereas the common known fungal genera were *Cryptococcus*, *Rhizosphaera*, *Mycopappus*, *Melampsora*, *Tetracladium*, *Phaeosphaeria*, *Mrakia*, *Venturia*, and *Leptosphaeria*. Both the climate and host-related factors might shape the fungal communities associated with the four Arctic plant species in this region. These results suggested the presence of an interesting endophytic fungal community and could improve our understanding of fungal evolution and ecology in the Arctic terrestrial ecosystems.

## Introduction

The Arctic is a unique area with extreme conditions (e.g., low temperatures, strong winds, permafrost, and long periods of darkness and light) that are challenging for most life forms. Microbial communities in the Arctic represent the largest reservoir of incompletely described biodiversity adapted to extreme conditions. An important component of Arctic microbial diversity is represented by fungi, and fungi in the Arctic can be found in diverse habitats, such as soil [1–3], ice [4, 5], cryoconite holes [6], moss [7], dead leaves [8], washed-up and trapped wood [9], and plant roots [10–18]. In recent years, Arctic microbial communities have been seriously threatened by climate change, as Arctic ecosystems are experiencing the greatest rates of climate warming on the planet and marked changes have already been observed in terrestrial Arctic ecosystems [19]. Understanding the effects of climate change on Arctic ecosystems may be enhanced by obtaining baseline data on the diversity of endophytic fungi in the region.

Endophytic fungi inhabit living tissues of plants at certain phases of their life cycle without causing apparent symptoms of infection [20]. These endophytic fungi may promote plant growth [21], affect plant resistance to abiotic (temperature, pH, and osmotic pressure) and biotic stresses (originating from bacteria, fungi, nematodes, and insects) [22], and decompose plant litter [23]. Fungal endophytes have been isolated from a variety of plant types (e.g., mosses, liverworts, hornworts, ferns, and seed plants) and are distributed world-wide [24–27]. However, the vast majority of fungal endophytes associated with Arctic vascular plants have yet to be adequately characterized.

The vegetation in the Arctic is characterized by the absence of trees and the presence of frost-tolerant plants that are challenged by several abiotic stresses (e.g., low temperatures, short growing season, high solar radiation, and drought) and that are highly adapted to their environment. Hitherto, the diverse fungal communities were found associated with roots of vascular plants in the Arctic [10–18] and the endophytic bacterial communities were also detected in Arctic vascular plants [28]. Only two studies reported on the endophytic fungi associated with above-ground plant tissues (i.e., leaves and stems) in the Arctic, including *Dryas octopetala* in Svalbard [24], *Huperzia selago*, *Picea mariana*, and *Dryas integrifolia* in the Canadian Arctic [29].

The Svalbard archipelago is located in the High Arctic and contains 173 vascular plant species, of which 167 are indigenous [30]. The species number in Svalbard is considerable lower than the 500 to 600 species in Tromsø, Norway (about 10 further south) [31]. The North Atlantic Current (the Gulf Stream) carries relatively warm water along the west coast of Svalbard and makes it possible for some non-Arctic plants to grow there in the summer. About 564 fungal species of 238 genera (excluding lichenized and lichenicolous species) have been reported in Svalbard [30], but the mycological information for Svalbard, especially with respect to endophytic fungi, is quite incomplete.

The 454 pyrosequencing technique significantly enhances the characterization of fungal diversity compared to traditional methods (e.g., pure culture techniques, clone library, or fingerprinting methods) and has proved useful for determining the composition of endophytic fungal communities [32]. In the present study, we used 454 pyrosequencing to investigate the fungal community associated with the leaves and stems of vascular plants in the High Arctic to address the following questions: (1) What fungal taxa exist within leaves and stems of vascular plants in the High Arctic? (2) What factors may shape the fungal communities associated with these four vascular plant species in this region?

## Materials and Methods

### Sample collection and ethics statement

**Fig 1:**
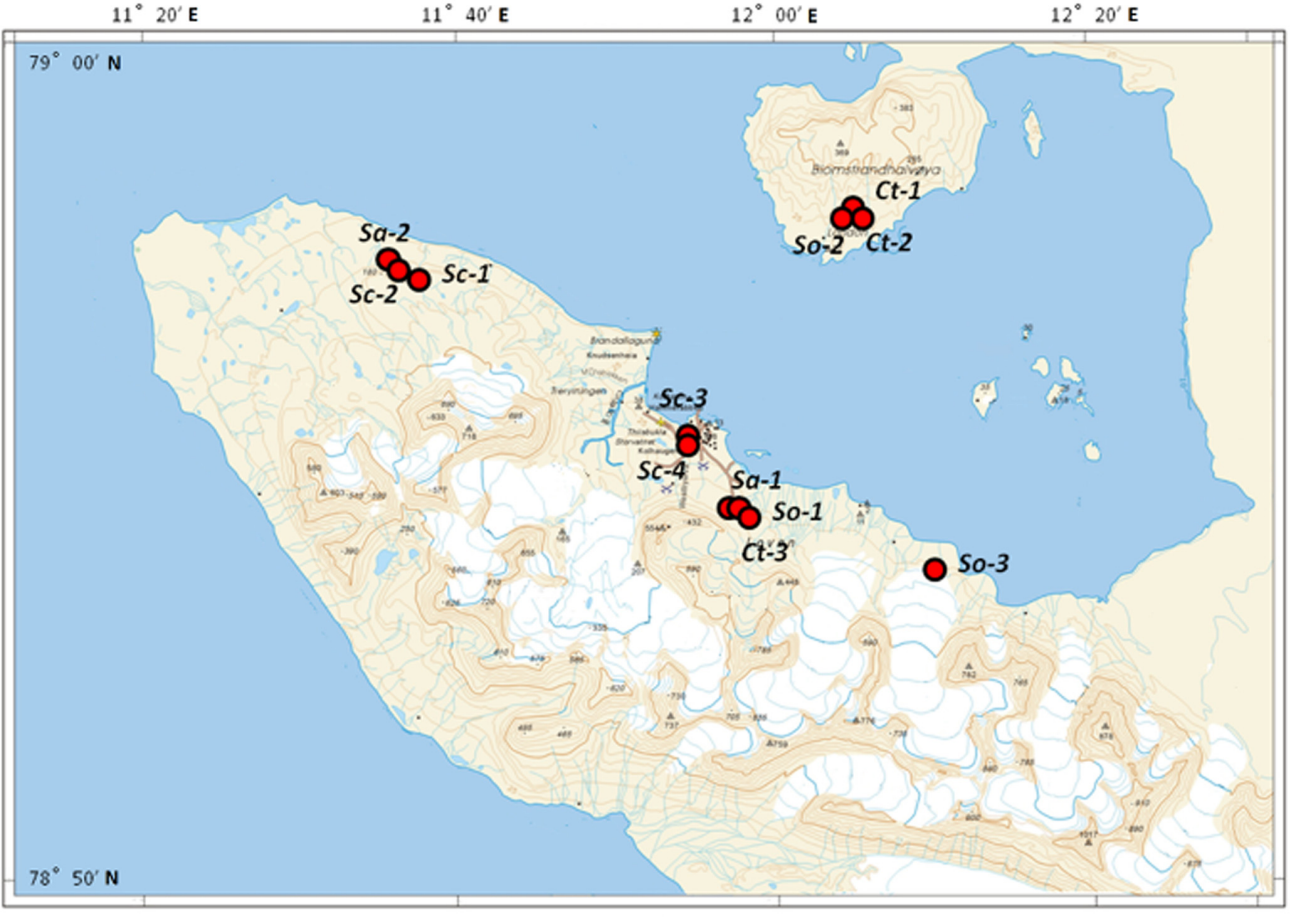
Map of Ny-Ålseund region in Svalbard (High Arctic) showing the sites where plant samples were collected for this study.

**Fig 2:**
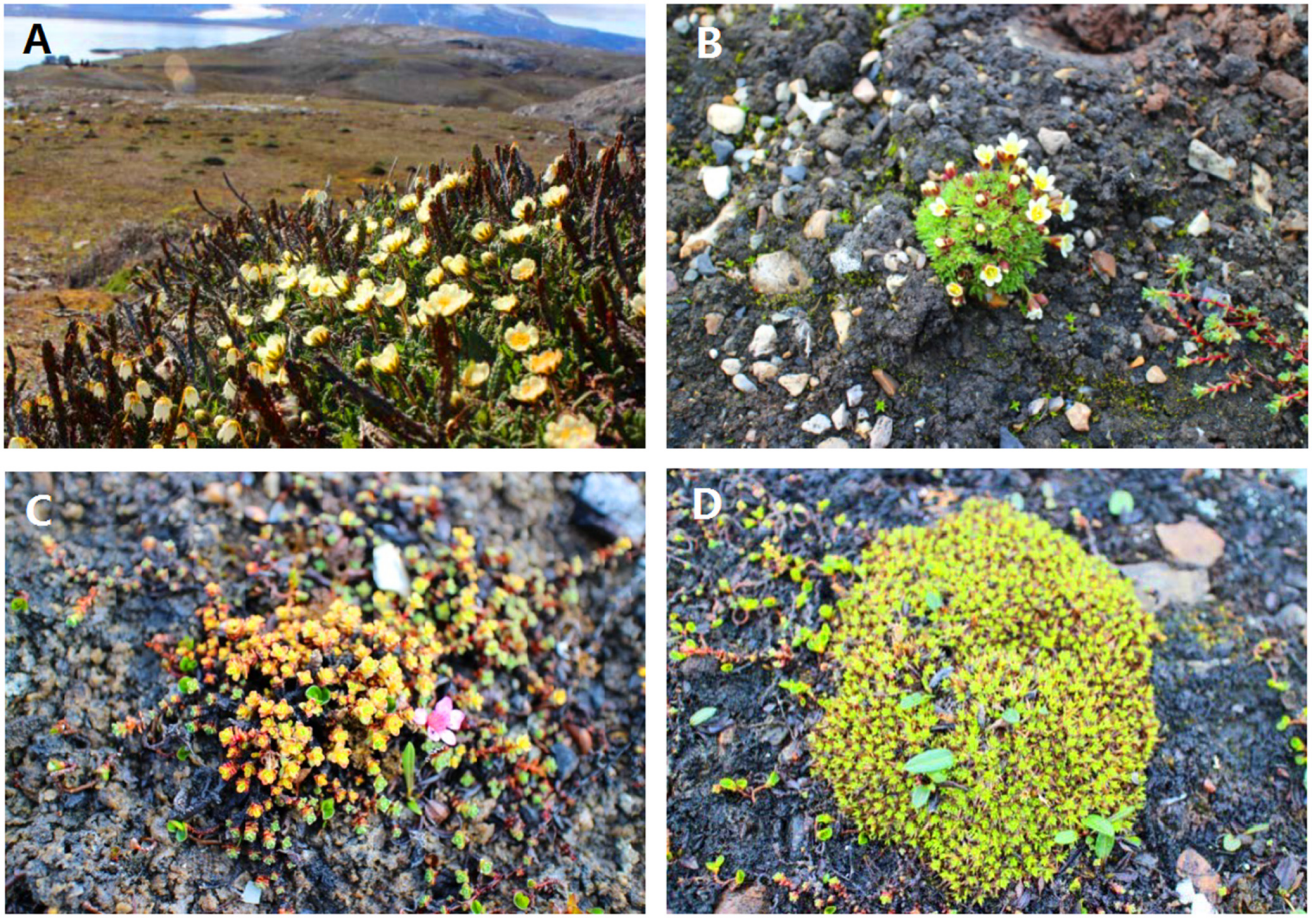
The four vascular plant species studied in the Ny-Ålesund region, Svalbard (High Arctic). A. *Cassiope tetragona*; B. *Saxifraga cespitosa*; C. *Saxifraga oppositifolia*; D. *Silene acaulis*.

The study area is located in the Ny-Ålesund region (78°55' N, 11°56' E), Spitsbergen, the largest island of the Svalbard archipelago. The Svalbard archipelago is geographically isolated from mainland Eurasia, which is entirely within the High Arctic. The climate is dominated by the island’s high latitude, but is moderated by the North Atlantic current, especially during winter. The Ny-Ålesund region is situated on the Brøgger peninsula (Brøggerhalvøya) and on the shore of Kongsfjorden Bay. The mean summer temperatures (June-August) in Ny-Ålesund was 3.6°C, the mean annual temperature was -6.0°C, and the mean annual precipitation was 371 mm, with only 78 mm falling during the three summer months [33]. Sampling was performed in the Ny-Ålesund region during China’s Arctic expedition in July 2013. The four common vascular plant species represented by 12 samples were collected in this area (Table 1, Figs 1 and 2). The sample collection was under ethical approval of the Svalbard Science Forum (Research in Svalbard ID 4951 & 7126) and the Chinese Arctic and Antarctic Administration (CAA), the State Oceanic Administration (SOA) of China.

**Table 1:** Information for the 12 vascular plant samples collected from the Ny-Ålesund region, Svalbard.

Sample code	Plant species	Coordinations	Altitude	Valid^a^	Trimmed^b^	OTU^c^	Chao1	Coverage	Shannon index (*H*’)
Ct-1	*Cassiope tetragona* (L.) D. Don.	78°57’58.73”N;12°03’33.88”E	45m	23204	11753	69	86(74,123)	99.8	2.73
Ct-2	*Cassiope tetragona* (L.) D. Don.	78°57’58.73”N;12°03’34.18”E	45m	16305	5597	84	105(90,156)	99.7	4.24
Ct-3	*Cassiope tetragona* (L.) D. Don.	78°54’40.26”N;11°56’59.12”E	75m	8570	4578	43	56(47,89)	99.7	2.83
Sc-1	*Saxifraga cespitosa* L.	78°57’54.52”N;11°35’05.38”E	17m	16174	8568	65	100(75,191)	99.8	3.81
Sc-2	*Saxifraga cespitosa* L.	78°57’37.76”N;11°36’10.25”E	33m	15744	4964	64	68(65,84)	99.8	3.92
Sc-3	*Saxifraga cespitosa* L.	78°55’40.09”N;11°54’31.84”E	10m	17281	9217	56	64(58,88)	99.9	2.82
Sc-4	*Saxifraga cespitosa* L.	78°55’39.99”N;11°54’32.25”E	11m	6043	4294	76	93(81,130)	99.6	3.83
So-1	*Saxifraga oppositifolia* L.	78°55’09.54”N;11°56’32.31”E	19m	9121	6250	87	98(90,129)	99.8	3.54
So-2	*Saxifraga oppositifolia* L.	78°57’58.70”N;12°03’34.39”E	45m	9356	6562	77	87(80,110)	99.7	4.11
So-3	*Saxifraga oppositifolia* L.	78°53’43.42”N;12°09’43.69”E	67m	13378	6389	45	47(45,60)	99.9	3.37
Sa-1	*Silene acaulis* (L.) Jacq.	78°55’05.81”N;11°56’36.35”E	19m	8219	3542	31	39(34,62)	99.7	2.24
Sa-2	*Silene acaulis* (L.) Jacq.	78°58’00.74”N;11°35’01.31”E	17m	7579	4977	31	45(33,98)	99.8	2.64

^a^Number of valid sequences.

^b^Number of trimmed sequences.

^c^Number of OTUs.

These four vascular plant species are not native in Svalbard. *Cassiope tetragona* (Arctic white heather; Ericaceae) is distributed in the High Arctic and northern Norway, grows on ridges and heaths, is often in abundant and forms a distinctive plant community. *Saxifraga cespitosa* (tufted saxifrage; Saxifragaceae) is common to many Arctic areas and appears further south in mountainous areas (e.g., the Alps, Norway, Iceland, Siberia, and western North America), and grows on ledges and in gravelly habitats. *Saxifraga oppositifolia* (purple saxifrage; Saxifragaceae) is very common all over the High Arctic (e.g., north Greenland and Svalbard), and is also found in some high mountain areas (e.g., northern Britain, the Alps, and the Rocky Mountains), and grows in all kinds of habitats, from sea level up to 4505 m a.s.l. *Silene acaulis* (moss campion; Caryophyllaceae) is common all over the High Arctic and tundra in the higher mountains of Eurasia and North America, and grows mainly in dry, gravelly localities, but also in damper places [31].

### DNA extraction

Before DNA extraction, plant tissues without roots were surface sterilized by immersion in 75% ethanol for 1 min, in 1% sodium hypochlorite for 2 min, and in 75% ethanol for 0.5 min. The surface-sterilized tissues were then rinsed with sterile water for 0.5 min and blotted dry with sterile filter paper. To test the effectiveness of surface sterilization, approximately 1 ml of the final rinse water of each sample was plated on potato dextrose agar (PDA). We cut the sterilized samples into small segments (2∼3 cm long stems with numbers of leaves) with sterilized scissors and then used a SuperFastPrep-1 Instrument (MP Biomedicals Co., Ltd.) to crush these segments. Genomic DNA was extracted from 36 segments of the 12 vascular plant samples (three segments for each sample) using a PowerSoil DNA Isolation Kit (MO BIO Laboratories, Inc.) according to the manufacturer’s instructions. The obtained DNA extracts were then used for PCR and sequencing analyses.

### 454 pyrosequencing

The fungal internal transcribed spacer (ITS, ITS1-5.8S-ITS2) of nuclear ribosomal DNA sequences was amplified by using a set of primers designed by adding a 10-nucleotide barcode to ITS1F and ITS4 primer sets [34]. The 20 μl reaction mixture contained 10 ng of template DNA, 4 μl of 5×buffer, 2 μl of 2.5 nM dNTP, 0.8 μl of Fastpfu Polymerase, 2 μM of each primer and ddH_2_O. The PCR amplification consisted of an initial denaturation at 95°C for 2 min; 30 cycles of denaturation at 95°C for 30 s, annealing at 55°C for 30 s, and extension at 72°C for 30 s; and a final extension at 72°C for 5 min. The PCR products were purified with an AxyPrepDNA Gel Extraction Kit (Axyen Scientific, Inc.) according to the manufacturer’s instructions. The purified PCR amplicons from each sample were mixed and then pyrosequenced by using the 454 GS FLX + Platform (Roche Applied Science, USA) at Majorbio Bio-Pharm Technology Co., Ltd., Shanghai, China. The raw sequence reads were deposited in the NCBI sequencing read archive (SRA) under accession no. SRP049024.

### Pyrosequencing data processing

The raw sequence data generated from pyrosequencing were processed with QIIME 1.8.0 software [35]. In brief, the sequence libraries were split and denoised to avoid diversity overestimation caused by sequencing errors. The sequence reads were processed by removing tags and primer, only accepting reads with an average quality score above 20 and read lengths longer than 200 bp. The sequences were also screened for chimeras, and putative chimeric sequences were removed. The operational taxonomic units (OTUs) were defined on the basis of a 97% similarity threshold. These OTUs were then used as a basis for calculating alpha-diversity and beta-diversity metrics using QIIME 1.8.0 software.

### Statistical analyses

Sequences representing the OTUs were subjected to BLASTn searches in GenBank (http://www.ncbi.nlm.nih.gov/genbank/) to determine their taxonomic affiliation. The following criteria were used to interpret the sequences of the GenBank database: for sequence identities ≥ 97%, the genus and species were accepted; for sequence identities between 95% and 97%, only the genus was accepted; and for sequence identities <95%, OTUs were labelled with the order, family, or phylum name or as ‘unassigned’. The OTU richness of each plant species according to three indices (Chao1, coverage and Shannon- index) was analyzed using the QIIME 1.8.0 software [35]. The abundance-based Bray-Curtis similarity coefficient was used to examine the dissimilarity of plant samples. The relationships among the fungal communities in the plant samples were analyzed using nonmetric multidimensional scaling (NMDS) with R 3.1.1 statistical software (http://www.r-project.org/). To determine the relationships between the endophytic fungal communities in the Arctic and those in non-Arctic environments, the 454 pyrosequencing data were downloaded from the NCBI-SRA database with the accession numbers PRJNA34493 & PRJDB2528) and were analyzed by principal coordinate analyses (PCoA) using the Metagenomics RAST server (MG-RAST, release 3.5) (http://metagenomics.anl.gov/). Network analysis using Gephi 0.8.2 software [36] was used to visualize all of the OTUs and to compare their abundance among the four Arctic plant species. The similarities among fungal communities from different plant species were estimated using Sorenson’s similarity coefficient (*CS*), which was calculated according to the formula: *CS = 2C/(A+B)*, where *A* and *B* are the OTU numbers in samples *A* and *B*, respectively, and *C* is the number of OTUs shared by the two species. *CS* values range from 0 (no similarity) to 1 (absolute similarity).

## Results

### Sequence data

The raw sequence data of the 12 samples consisted of 184,242 reads, of which 76,691 were retained after sequences with different tags at each end were removed for quality filtering and denoising. After singletons, chimeric sequences, and OTUs assigned to nontarget organisms were removed, 250 fungal OTUs of 76,691 reads were included in the final matrix. The number of OTUs per plant sample ranged from 31 to 87 (Table 1).

### Fungal diversity and community structure

Based on BLASTn searches in GenBank, the OTUs were identified at different levels of taxonomic precision. The information for the 250 OTUs was presented in S1 and S2 Tables. Among the 250 OTUs, 127 had sequences with high similarity (>97% matching) to those in GenBank; most of those from GenBank had been previously reported from the Arctic (e.g., Svalbard, North American Arctic) and areas near the Arctic (e.g., regions in Norway, Finland, Austria, Sweden, Switzerland, Russia, Portugal, Germany, and northern USA). Of these 127 OTUs, 56 had sequences that were similar to those from fungi associated with plant tissues (i.e., wood stumps, roots, stems, and leaves) and 46 has sequences that were similar to those from fungi living in soils. The other 123 OTUs had matching sequences with similarity below 97%, and these may belong to undiscovered fungal species.

**Fig 3:**
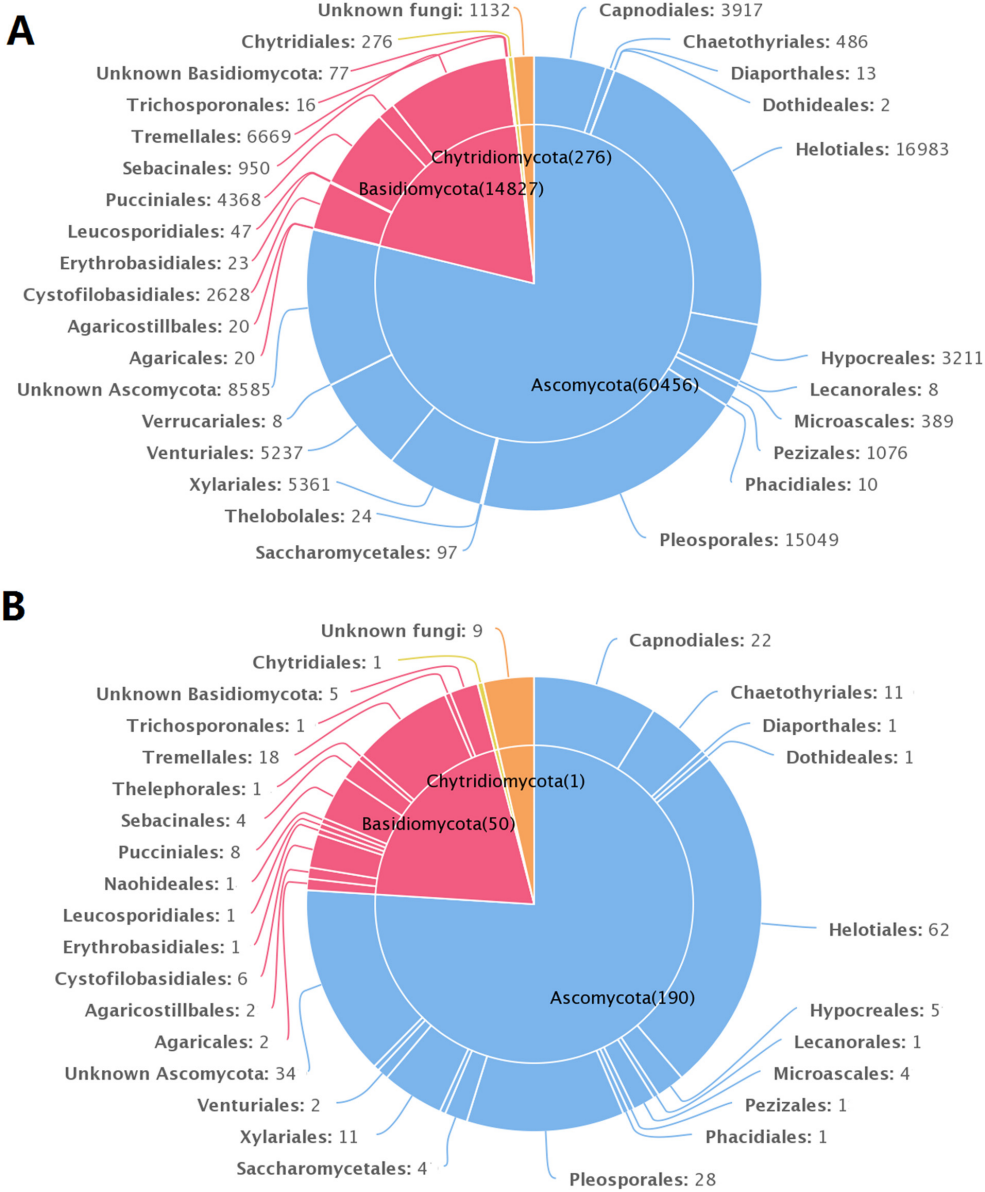
The pie charts showing the taxonomic distribution of sequences and operational taxonomic units (OTUs). (A) The taxonomic distribution of 76,691 sequences that were primarily conducted at the order level. (B) The taxonomic distribution of the 250 OTUs primarily at the order level.

The most precise taxonomic level to which the 250 OTUs could be assigned was to species for 41 OTUs, to genus for 64 OTUs, to family for 53 OTUs, to order for 49 OTUs, to class for 3 OTUs, to phylum for 30 OTUs, and to unknown fungi for 7 OTUs. These OTUs spanned 3 phyla, 14 classes, 28 orders, 41 families, 51 genera, and 31 species (Table 2 and S1 Table). Members of Ascomycota were more frequently observed than those of Basidiomycota and Chytridiomycota from all four plant species. Dothideomycetes, Leotiomycetes, Tremellomycetes and Sordariomycetes were the major classes, in terms of number of reads and OTUs (Table 2). The Ascomycota included 16 orders with Helotiales and Pleosporales being the most abundant and diverse. The Basidiomycota included 11 orders, with Tremellales being the most abundant and diverse, followed by Pucciniales and Cystofilobasidiales (Fig 3). However, the distributions of OTUs among orders were not same for all four vascular plant species (Table 2).

**Table 2:** An overview of the taxonomic composition of endophytic fungal communities found in four plant species in the Ny-Ålesund region, Svalbard.

	% of reads	% of OTUs	*Cassiope tetragona*	*Saxifraga cepitosa*	*Saxifraga oppositifolia*	*Silene acaulis*
**Ascomycota**	78.83	76.00	98.23	69.37	70.23	78.34
**Dothideomycetes**	**35.76**	**23.20**	**39.38**	**28.81**	**26.68**	**68.88**
Capnodiales	5.11	8.80	9.55	1.75	6.96	0.15
Dothideales	0.01	0.40	0.01	0.01	-	-
Hysteriales	4.19	2.00	0.40	0.06	0.91	34.42
Pleosporales	19.62	11.20	5.55	26.99	18.80	34.30
Venturiales	6.83	0.80	23.87	-	0.01	0.01
**Eurotiomycetes**	**0.64**	**4.80**	**1.75**	**0.38**	**0.03**	**-**
Chaetothyriales	0.63	4.40	1.75	0.37	0.01	-
Verrucariales	0.01	0.40	-	0.01	0.02	-
**Leotiomycetes**	**22.18**	**25.60**	**11.41**	**38.60**	**17.09**	**9.34**
Helotiales	22.14	24.80	11.39	38.53	17.04	9.33
Thelebolales	0.03	0.40	0.01	0.06	0.02	-
Phacidiales	0.01	0.40	0.01	0.01	0.03	0.01
**Lecanoromycetes**	**0.01**	**0.40**	**-**	**0.03**	**-**	**-**
Lecanorales	0.01	0.40	-	0.03	-	-
**Saccharomycetes**	**0.13**	**1.60**	**0.16**	**0.20**	**0.04**	**0.02**
Saccharomycetales	0.13	1.60	0.16	0.20	0.04	0.02
**Sordariomycetes**	**7.52**	**6.40**	7.71	**0.73**	**20.17**	**0.04**
Diaporthales	0.02	0.40	0.06	-	-	-
Microascales	0.51	1.60	-	0.43	1.42	-
Xylariales	6.99	4.40	7.65	0.30	18.75	0.04
**Pezizomycetes**	**1.40**	**0.40**	**4.71**	**-**	**0.20**	**0.05**
Pezizales	1.40	0.40	4.71	-	0.20	0.05
**Unknown Ascomycota**	**11.19**	**13.60**	**33.11**	**0.62**	**6.02**	**0.01**
**Basidiomycota**	**19.36**	**20.00**	**1.44**	**30.2**	**26.55**	**14.40**
**Agaricomycetes**	**1.28**	**2.80**	**0.95**	**1.31**	**0.01**	**4.77**
Agaricales	0.03	0.80	-	0.07	-	-
Sebacinales	1.24	1.60	0.95	1.24	-	4.77
Thelephorales	0.01	0.40	-	-	0.01	-
**Agaricostilbomyces**	**0.03**	**0.80**		**0.05**	**0.01**	**0.06**
Agaricostillbales	0.03	0.80	-	0.05	0.01	0.06
**Cystobasidiomycetes**	**0.04**	0.80	**-**	**-**	0.16	**-**
Erythrobasidiales	0.03	0.40	-	-	0.12	-
Naohideales	0.01	0.40	-	-	0.04	-
**Microbotryomycetes**	**0.06**	**0.40**	-	**0.04**	-	**0.43**
Leucosporidiales	0.06	0.40	-	0.04	-	0.43
**Pucciniomycetes**	**5.70**	**3.20**	**-**	**2.83**	18.77	-
Pucciniales	5.70	3.20	-	2.83	18.77	-
**Tremellomycetes**	**12.15**	**10.00**	**0.18**	**26.04**	**7.55**	**9.14**
Cystofilobasidiales	3.43	2.40	0.02	4.93	6.62	0.21
Tremellales	8.70	7.20	0.15	21.11	0.86	8.93
Trichosporonales	0.02	0.40	0.01	-	0.07	-
**Unknown Basidiomycota**	**0.10**	**2.00**	**0.31**	**-**	0.05	**-**
**Chytridiomycota**	**0.36**	0.40	**-**	**-**	1.44	-
**Chytridiomycetes**	**0.36**	**0.40**	**-**	**-**	**1.44**	**-**
Chytridiales	0.36	0.40	-	-	1.44	-
**Unknown fungi**	**1.48**	**3.60**	**0.35**	**0.35**	**1.79**	**7.25**

The second and third columns indicate the percentage of total OTUs and the total number of reads across the host species, respectively. The last four columns provide a taxonomic overview of the fungal communities found in each of the four plant species, which are represented as the percentage of sequence reads.

The fungal genera that were commonly detected (>1000 reads) were *Cryptococcus* (6304 reads, 9 OTUs), *Rhizosphaera* (5226 reads, 1 OTU), *Mycopappus* (4493 reads, 2 OTUs), *Melampsora* (4356 reads, 7 OTU), *Tetracladium* (3696 reads, 11 OTUs), *Phaeosphaeria* (2551 reads, 3 OTUs), *Mrakia* (2422 reads, 4 OTUs), *Venturia* (1774 reads, 5 OTUs), and *Leptosphaeria* (1340 reads, 5 OTUs). The fungal species that were commonly detected (>200 reads) were *Rhizosphaera macrospora* (5226 reads), *Phaeosphaeria triglochinicola* (1844 reads), *Leptosphaeria pedicularis* (1073 reads), *Venturia alpina* (1045), *Phoma herbarum* (833 reads), *Mrakia frigida* (779 reads), *Melampsora epitea* (549 reads), *Tetracladium furcatum* (401 reads), *Monodictys arctica* (374 reads), *Venturia minuta* (347 reads), *Eleutheromyces subulatus* (225 reads), and *Mycoarthris corallines* (201 reads) (S1 Table).

### Similarity of endophytic fungal communities

Chao1, coverage, and Shannon diversity indices were used to evaluate and compare the diversity of the fungal communities among the plant samples (Table 1). The coverage ranged from 99.7 to 99.9%, indicating that pyrosequencing captured the dominant phylotypes. The Shannon Index (*H*’ = 0.24–4.24) indicated that diversity vary among the 12 plant samples.

**Fig 4:**
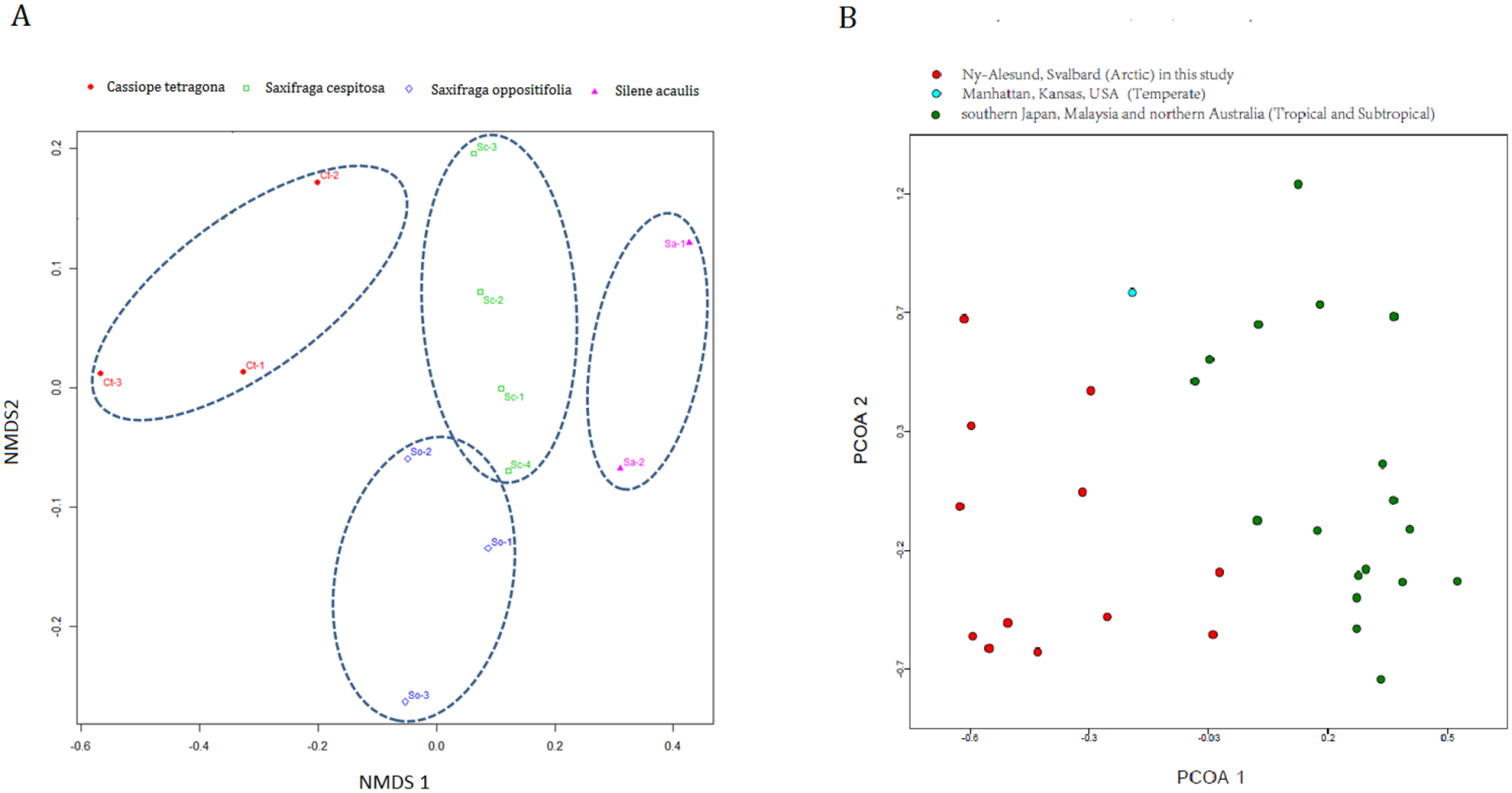
**A. Nonmetric multidimensional scaling (NMDS) ordination based on Bray-Curtis-transformed abundance data for OTUs**. Each point corresponds to a sample. The different colors/symbols represent the four vascular plant species. B. Principal coordinate analysis (PCoA) of the 12 samples in this study (red) compared to 19 samples from other environments (other colors).

The NMDS analyses indicated that the fungal communities are host specific but not site specific. No spatial structuring of the fungal communities among the different sites was evident in the NMDS diagram (Fig 4A). The PCoA analyses indicated that the endophytic fungal communities in the Arctic differ from endophytic fungi associated with tree leaves in tropical and subtropical forests (southern Japan, Malaysia, and northern Australia) and in the temperate region (Manhattan, Kansas, USA) (Fig 4B).

Sorenson’s similarity coefficients for endophytic fungal community composition were low among the four plant species (Table 3). The highest similarity (0.66) was between *Saxifraga cepitosa* and *Saxifraga oppositifolia*, and the lowest similarity (0.32) was between *Cassiope tetragona* and *Saxifraga cepitosa*. These low Sorenson’s similarity coefficients indicated that the fungi have different distributions among the plant species and perhaps some degree of host specificity.

**Table 3:** Sorenson’s similarity coefficients for endophytic fungal communities among the four plant species in the Ny-Ålesund region.

Plant species	*Cassiope tetragona*	*Saxifraga cepitosa*	*Saxifraga oppositifolia*
*Cassiope tetragona*	-	-	-
*Saxifraga cepitosa*	0.32	-	-
*Saxifraga oppositifolia*	0.42	0.66	-
*Silene acaulis*	0.35	0.40	0.37

**Fig 5:**
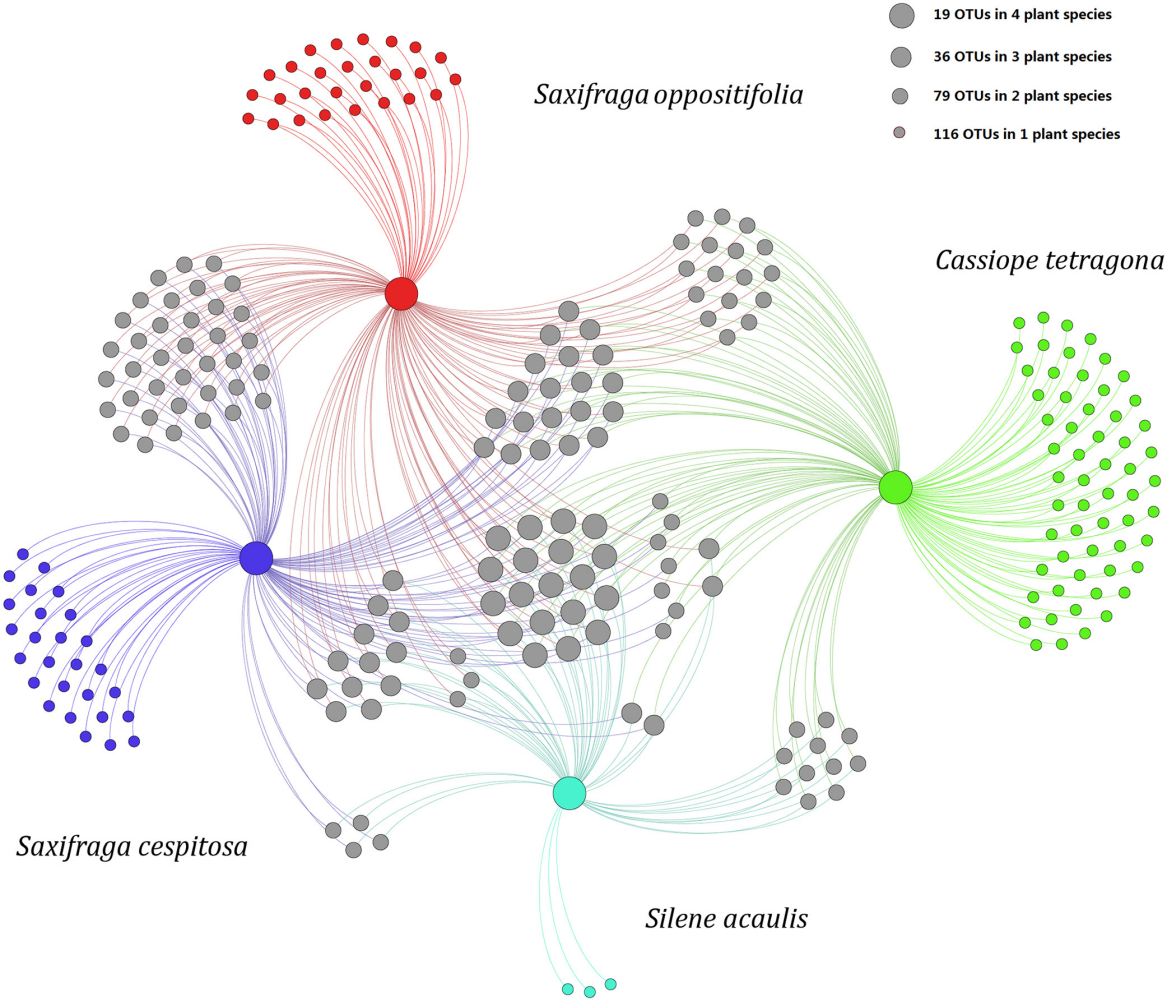
A Gephi network visualizing the 250 OTUs and demonstrating the number of shared OTUs among the four Arctic plant species.

To better elucidate the distribution of OTUs among the four plant species, a network analysis was performed (Fig 5). The network diagram, which illustrated OTU partitioning among the plant species, revealed many unique OTUs that belonged to only one plant species. Among the 250 OTUs, 116 belonged to only one plant species, 79 to two plant species, 36 to three plant species, and 19 to all four plant species.

## Discussion

The significance of endophytic fungi in leaves and stems of Arctic vascular plants was unclear, mainly because data on the fungal species in this habitat were limited. This study was the first to use high-throughput pyrosequencing in order to comprehensively analyze the fungal communities associated with vascular plants in the High Arctic.

### The endophytic fungal communities in Arctic plants were highly diverse

Despite Svalbard’s geographic isolation and its extreme environmental conditions, diverse endophytic fungi were found in this region. The wide range of Shannon diversity indices (*H*’ = 2.24–4.24) suggested the presence of a complex fungal network within Arctic plant tissues. Using traditional culture-based methods, Rosa et al. [27] reported low diversity (*H*’ = 1.10–2.02) for fungal communities in an Antarctic vascular plants (*Colobanthus quitensis*; Caryophyllaceae) and relatively low diversity for fungal communities (*H*’ = 1.25–2.69) was also found in the leaves and stems of five dominant plant species (i.e., *Rhododendron* spp., *Quercus pannosa*, and *Quercus spinosa*) collected from the Baima Snow Mountain (altitude 4,000–4,300 m) in China [37]. Using 454 pyrosequencing, however, high diversity for fungal communities (*H*’ = 2.75–10.38) were found in the leaves of temperate *Quercus macrocarpa* from Manhattan (Kansas, USA) [32]. The high sensitivity of 454 pyrosequencing enables the detection of rare species and 454 pyrosequencing thereby provides more detailed information on fungal diversity than conventional sequencing methods. In the extreme environment of the Arctic, a diverse community of endophytic fungi may benefit host plants by providing nutrients and increasing stress resistance.

Our results indicated that members of Ascomycota are more common than those of Basidiomycota and Chytridiomycota as endophytes of the four plant species in the Arctic. The fungal taxa of the Chytridiomycota have rarely been detected as endophytes but have been observed in phyllosphere fungal communities [38]. Members of Basidiomycota were detected more often than those of Ascomycota, Zygomycota, or Glomeromycota in the root-associated fungal communities from Svalbard (e.g., *Bistorta vivipara*, *Salix Polaris*, and *Dryas octopetala*) [18]. These data indicated that fungal communities in leaves and stems are distinct from those associated with plant roots in the Arctic.

Among the 51 known genera reported in this study, *Articulospora*, *Cladosporium*, *Fusarium*, *Phaeosphaeria*, and *Phoma* have previously been isolated from Arctic plants (e.g., *Dryas octopetala* in Svalbard, *Huperzia selago*, *Picea mariana*, and *Dryas integrifolia* in the Canadian Arctic) [24, 30], whereas *Cadophora*, *Helgardia*, *Herpotrichia*, and *Oculimacula* have been isolated from Antarctica vascular plants (e.g., *Deschampsia antarctica* and *Colobanthus quitensis*) [26, 27]. In addition, the following fungal genera detected in the present study were previously detected as plant endophytes in non-Polar regions: *Acremonium*, *Chloridium*, *Ilyonectria*, *Monodictys*, *Cladosporium*, *Phoma*, *Seimatosporium*, *Cryptococcus*, *Fusarium*, *Leptodontidium*, *Leptosphaeria*, *Pestalotiopsis*, *Rhinocladiella*, *Rhizosphaera*, and *Venturia* [39–45]. Interestingly, some genera detected have been observed in other habitats. For example, members of genera *Cryptococcus*, *Dioszegia*, *Leucosporidiella*, *Komagataella*, *Metschnikowia*, *Mrakia*, *Pichia*, *Udeiomyces*, *Sporobolomyces*, and *Trichosporon* were extremophilic yeasts that exist frequently in the Arctic soils [46].

Among the 31 known fungal species detected in this study, most were originally reported in other habitats and were reported here for the first time to be endophytes in leaves and stems of Arctic plants. For examples, *Monodictys arctica* was previously detected in the roots of *Saxifraga oppositifolia* collected in the Canadian High Arctic [47], and *Phoma herbarum* was a widespread saprophyte and pathogen of plants and animals and has been found in diverse environments including Antarctica [48]. These results indicated the presence of specific psychrophilic and psychrotrophic fungi in various habitats in the cold ecosystems. Furthermore, the wide distribution of these fungi suggested that they may be capable of long-distance dispersal. A substantial portion of the endophytic fungi detected in this study were unclassified and might be new and possibly indigenous species in the Svalbard.

### The endophytic fungal communities in Arctic plants were shaped by the Arctic climate

Understanding similarities and differences in fungal communities among plant samples could reveal key factors that shape the fungal communities in plants. In this study, the major class detected was Dothideomycetes, followed by Leotiomycetes, Tremellomycetes and Sordariomycetes. These data were in agreement with a previous study of boreal and Arctic endophytes of plants (*Huperzia selago*, *Picea mariana*, and *Dryas integrifolia*) in Canada, in which Dothideomycetes, Sordariomycetes, Chaetothyriomycetidae, Leotiomycetes, and Pezizomycetes were the major classes [24]. In tropical and temperate plants, in contrast, the major class of endophytes was Sordariomycetes, followed by Dothideomycetes and Leotiomycetes [25, 49, 50].

Helotiales, Pleosporales, Capnodiales, and Tremellales were the major orders detected in the present study, and this differed from the orders of endophytic fungi previously detected in non Arctic plants. Based on 454 pyrosequencing of fungi from temperate plants, Pleosporales, Capnodiales, and Erysiphales were the common orders from leaves of *Quercus macrocarpa* [32], and Taphrinales, Capnodiales, Dothideales, Tremellales, and Sporidiobolales were the common orders from leaves of *Fagus sylvatica* [51].

The PCoA diagram also showed that the fungal communities detected in leaves and stems of plants in the Ny-Ålesund region differ from endophytic fungi associated with plant leaves in tropical and subtropical forests (southern Japan, Malaysia and northern Australia) and in a temperate region (Manhattan, Kansas, USA). Taken together, these results indicated that the endophytic fungal communities in the Arctic differ from those in temperate and tropical regions, suggesting that the endophytic fungal communities in Arctic plants may be shaped by Arctic climate conditions. Similarly, Fisher et al. [29] reported that fungal endophyte assemblages of *Dryas octopetala* differ between Spitsbergen (Svalbard) and the Swiss Alps [29]. Petrini [52] proposed that climatic conditions might greatly influence the colonization of plants by endophytic fungi.

### The endophytic fungal communities in Arctic plants showed a degree of host specificity

In the present study, the fungal communities differed among the four plant species at the order level (Table 2). The NMDS and network analyses of OTUs demonstrated a specific association between OTUs and host species (Figs 4 and 5). These results suggested some degree of host-specificity but no spatial structuring for the endophytic fungal communities in this region, even though these four plants inhabit a similar stressful environment. Previous studies also reported that the composition of fungal communities associated with above-ground plant tissues depend on the host species [24, 53, 54].

Most endophytic fungi colonized the host plant by growing locally in certain plant tissue or organs (e.g., leaves or stems). The four plant species sampled in the present study belonged to different plant families and differed greatly in leaf and stem morphology, structure, and tissue texture and elasticity. For example, the leaves of *Silene acaulis* were narrow, thin, and soft, whereas the leaves of *Cassiope tetragona* were grooved, relatively thick, and tough. It was possible that the fungal communities in the different plant species have adapted to different types of leaves and stems.

In summary, our pyrosequencing results indicated that the leaves and stems of four Arctic plants (*Cassiope tetragona*, *Saxifraga cespitosa*, *Saxifraga oppositifolia*, and *Silene acaulis*) harbor many fungal species, some of which may be endemic and/or indigenous fungal species. Both the Arctic climate and host-related factors might shape the fungal communities associated with these four vascular plant species in this region. Given that the Arctic has been particularly susceptible to climate change [19], the endophytic fungal communities in the Ny-Ålesund region may be useful for study the effect of climate change on Arctic terrestrial ecosystems.

## Supporting Information

S1 Table Overview of the 250 OTUs found in the 12 plant samples, including their frequency, number of reads and BLASTn top hits with accession numbers in GenBank. (PDF)

S2 Table Distribution of the 250 OTUs found in the 12 plant samples, including their number of sequences. (PDF)
