# Supplementary material for: Endophytic Fungal Communities Associated with Vascular Plants in the High Arctic Zone Are Highly Diverse and Host-Plant Specific
Source: PLoS One. 2015 Jun 12;10(6):e0130051. doi: 10.1371/journal.pone.0130051 (PMC4466372; doi:10.1371/journal.pone.0130051)
Supplement: S1 Table — (PDF) [file pone.0130051.s001.pdf]

S1 Table. Overview of the OTUs found in the 12 plant samples, including their frequency, number of reads and BLASTN top hits with accession numbers in GenBank.

**OTU ID**	**Freq.**	**Reads**	**phylum**	**Order**	**Family**	**Identification**	**Close GenBank match / Accession**	**Similarity**	**Originally reported habitat**
OTU 1	10	779	Basidiomycota	Cystofilobasidiales	Cystofilobasidiaceae	*Mrakia frigida*[a]	*Mrakia frigida* AJ866977	**99%(476/480)**	Sediment in Russia (Siberia)
OTU 2	4	5226	Ascomycota	Venturiales	Venturiaceae	*Rhizosphaera macrospora*[a]	*Rhizosphaera macrospora* AF462431	**97%(466/481)**	Fungal conifer pathogens (*Abies alba*) in France
OTU 3	2	5	Ascomycota	Helotiales	Unassigned	Helotiales sp.	Uncultured fungus clone GU174334	94%(492/521)	Forest floor (*Acer saccharum*) in USA: Michigan, Manistee National Forest (43.6687N; 86.1431W)
							Uncultured Helotiales clone EU726288	93%(433/465)	Plant (*Pinus ponderosa*) in California woodland
OTU 4	2	5	Basidiomycota	Agaricostilbales	Agaricostilbaceae	Agaricostilbaceae sp.	*Bensingtonia sorbi* AY233343	90%(415/463)	Unreported
OTU 5	1	16	Ascomycota	Helotiales	Unassigned	Helotiales sp.	Uncultured *Tetracladium* clone KC694156	93%(428/462)	Plant roots (*Triticum aestivum*) in Sweden
OTU 6	6	469	Ascomycota	Xylariales	Amphisphaeriaceae	*Seeimatosporium* sp.[b]	*Seimatosporium walkeri* JN871207	95%(509/534)	Plant (*Eucalyptus* sp.) in Australia (Victoria)
OTU 7	1	11	Ascomycota	Chaetothyriales	Unassigned	Chaetothyriales sp.	Uncultured fungus clone KF274490	88%(343/388)	Wood stump (*Picea abies*) in Finland
							Rhinocladiella sp. EU139138	89%(336/379)	Lichen Umbilicariales in China (Hebei)
OTU 8	2	4	Basidiomycota	Tremellales	Unassigned	Tremellales sp.	Uncultured fungus clone JX383662	89%(337/379)	Soil in USA
							Uncultured Tremellales clone GU327507	96%(238/249)	Mycorrhizal seedling of *Epipactis helleborine* in Czech
OTU 9	3	142	Ascomycota	Chaetothyriales	Herpotrichiellaceae	Herpotrichiellaceae sp.	Herpotrichiellaceae sp. KF636410	94%(306/327)	Liverwort (*Barbilophozia hatcheri*) in Antarctica
OTU 10	1	70	Ascomycota	Unassigned	Unassigned	Ascomycota sp.	Uncultured fungus clone KC965841	95%(390/410)	Arctic soil in USA (69.67N; 148.72W)
							Cyphellophora laciniata NR121335	86%(361/419)	Skin of Homo sapiens in Switzerland
OTU 11	4	30	Basidiomycota	Unassigned	Unassigned	Basidiomycota sp.	Uncultured fungus clone KF800411	84%(467/556)	House dust in USA (Missouri, Kansas City)
							Mrakia blollopis AB916516	83%(447/541)	Arctic bird feather in Norway
OTU 12	2	2	Basidiomycota	Tremellales	Unassigned	Tremellales sp.	Uncultured fungus clone KF274456	95%(352/369)	*Picea abies* stumps in Finland
							Uncultured Tremellales clone JF449484	**99%(308/309)**	Beech litter in Austria (Tyrol:Achenkirch)
OTU 13	1	2	Basidiomycota	Unassigned	Unassigned	Basidiomycota sp.	Uncultured fungus clone KF274089	87%(432/496)	*Picea abies* stumps in Finland
							Rhodotorula sp. FN548148	87%(433/497)	Living surface-sterilised leaves of Fagus sylvatica in Germany

OTU ID	Freq.	Reads	phylum	Order	Family	Identification	Close GenBank match / Accession	Similarity	Originally reported habitat
									(City of Greifswald, nature conservation area Elisenhain)
OTU 14	1	4	Ascomycota	Chaetothyriales	Herpotrichiellaceae	Herpotrichiellaceae sp.	*Cladophialophora minutissima* EF016383	94%(422/447)	Unreported
OTU 15	2	152	Ascomycota	Pleosporales	Leptosphaeriaceae	*Leptosphaeria* sp.[b]	Uncultured *Leptosphaeria* clone EU852362	**97%(265/272)**	Plant (*Fraxinus excelsior*) in Sweden
OTU 16	1	17	Ascomycota	Unassigned	Unassigned	Ascomycota sp.	Uncultured soil fungus clone GU083291	93%(298/322)	Soil in USA (Alaska)
							Uncultured Sebacinales clone FJ788795	87%(317/364)	Mycorrhizal root section of *Pterygodium caffrum*
OTU 17	1	3	Ascomcyota	Unassigned	Unassigned	Ascomycota sp.	Uncultured fungus clone KC965419	99%(465/468)	Arctic soil in USA (69.67N; 148.72W)
							Pseudocercospora nephrolepidicola HQ599590	85%(347/406)	On leaves of *Nephrolepis falcata* in Australia
OTU 18	7	247	Basidiomycota	Cystofilobasidiales	Cystofilobasidiaceae	*Mrakia* sp.[b]	Uncultured *Mrakia* isolate GU931743	**99%(514/516)**	House dust in Canada (45.2556N; 75.9204W)
OTU 19	3	57	Ascomycota	Helotiales	Rutstroemiaceae	Rutstroemiaceae sp.	*Lambertella* sp. AB705255	94%(485/517)	Unreported
OTU 20	2	129	Ascomycota	Helotiales	Unassigned	*Tetracladium* sp.[b]	Uncultured *Tetracladium* clone KC694156	**97%(494/507)**	Roots of *Triticum aestivum* in Sweden
OTU 21	1	4	Ascomycota	Helotiales	Unassigned	Helotiales sp.	Uncultured mycorrhizal fungus AB669661	**99%(467/472)**	Unreported
							Uncultured Helotiales clone KC455325	**99%(449/451)**	Root system in Arctic tundra (USA: Anaktuvuk River Fire, AK)
OTU 22	7	624	Ascomycota	Unassigned	Unassigned	*Oleoguttula* sp.[b]	Uncultured fungus clone KC966025	96%(496/515)	Arctic soil in Canada (76.23N; 119.30W)
							Oleoguttula mirabilis KF309972	96%(448/468)	Unreported
OTU 23	1	3	Basidiomycota	Tremellales	Unassigned	*Cryptococcus* sp.[b]	Uncultured Basidiomycota clone GU328524	97%(478/494)	Oe layer in Forest
							Cryptococcus sp. HG324303	96%(440/456)	Soil in Germany (Biosphere area Schwaebische Alb)
OTU 24	3	10	Ascomycota	Helotiales	Helotiaceae	*Articulospora* sp.[b]	*Articulospora tetracladia* EU998929	95%(315/332)	Unreported
OTU 25	2	61	Ascomycota	Unassigned	Unassigned	Ascomycota sp.	Uncultured ascomycete clone AM901772	91%(464/510)	House dust in Finland
OTU 26	5	534	Ascomycota	Capnodiales	Unassigned	Capnodiales sp.	Uncultured fungus clone KC965669	**98%(480/490)**	Arctic soil in USA (69.15N; 148.85W)
							Penidiella ellipsoidea JF499843	91%(471/518)	Leaf bracts (*Phaenocoma prolifera*) in South Africa
OTU 27	2	37	Ascomycota	Microascales	Halosphaeriaceae	*Monodictys arctica*[a]	Uncultured fungus clone GU817175	**97%(239/302)**	Root systems of Bistorta vivipara in Norway (Svalbard Midtre Lovenbreen) (78.90555N; 12.08450E)
							Monodictys arctica EU686522	**97%(291/299)**	Roots of *Saxifraga oppositifolia* in Canadian High Arctic
OTU 28	10	1968	Basidiomycota	Tremellales	Unassigned	*Cryptococcus* sp.[b]	Uncultured *Cryptococcus* clone KC753413	**99%(491/496)**	Plant (*Triticum aestivum*) stem in Sweden

OTU ID	Freq.	Reads	phylum	Order	Family	Identification	Close GenBank match / Accession	Similarity	Originally reported habitat
OTU 29	1	7	Unassigned	Unassigned	Unassigned	Fungus sp.	Uncultured fungus clone KF297272	**99%(462/467)**	Arctic soil in Canada (76.23N; 119.30W)
OTU 30	5	277	Ascomycota	Pleosporales	Melanommataceae	*Herpotrichia* sp.[b]	Fungal sp. KF527818	96%(368/384)	Plant (*Carex bigelowii*) root in Finland
							Herpotrichia juniperi JX981496	96% (334/348)	Plant in Poland
OTU 31	8	258	Basidiomcyota	Tremellales	Unassigned	Badidiomycota sp.	Uncultured *Cryptococcus* clone KC753413	**99%(213/216)**	Plant stem (*Triticum aestivum*) in Sweden
OTU 32	6	104	Ascomycota	Capnodiales	Davidiellaceae	*Cladosporium* sp.[b]	*Cladosporium* sp. KF367501	**99%(561/562)**	Untreated drinking water sources in Portugal
OTU 33	3	455	Ascomycota	Helotiales	Unassigned	Helotiales sp.	Uncultured *Helicodendron* clone GU931747	92%(347/376)	House dust in Canada (45.2556 N, 75.9204W)
OTU 34	3	629	Ascomycota	Capnodiales	Unassigned	Capnodiales sp.	*Catenulostroma protearum* GU214628	89%(511/571)	Plant (*Hakea sericea*) in South Africa
OTU 35	2	13	Ascomycota	Chaetothyriales	Unassigned	Chaetothyriales sp.	Uncultured fungus clone KC965234	96%(494/513)	Arctic soil in Canada (76.23N; 119.30W)
							Herpotrichiellaceae sp. JQ272383	87%(473/541)	USA (GRSMNP, Laurel Falls)
OTU 36	5	201	Ascomycota	Helotiales	Hyaloscyphaceae	*Mycoarthris corallinus*[a]	Uncultured Helotiales isolate GU931728	**97%(487/500)**	House dust in Canada (45.2556N; 75.9204W)
							Mycoarthris corallinus AF128440	**97%(460/473)**	Unreported
OTU 37	2	42	Ascomycota	Capnodiales	Teratosphaeriaceae	Teratosphaeriaceae sp.	Uncultured fungus clone KF296855	**99%(413/417)**	Arctic soil in Canada (78.78N; 103.55W)
							Teratosphaeria jonkershoekensis EU707864	91%(402/441)	Plant (*Protea* sp.) leaf in South Africa
OTU 38	2	435	Ascomycota	Helotiales	Unassigned	*Tetracladium* sp.[b]	Uncultured *Tetracladium* clone GU327472	**97%(517/533)**	Mycorrhizal seedling (Epipactis atrorubens) in Czech (Lednice)
OTU 39	7	881	Ascomycota	Pleosporales	Unassigned	Pleosporales sp.	Fungal sp. JX978255	**97%(420/431)**	2 or 3 year-old twig tissue from adult narrowleaf cottonwood growing in a common garden
							Pleosporales sp. AB751503	96% (394/411)	Unreported
OTU 40	2	342	Basidiomycota	Sebacinales	Sebacinaceae	*Sebacina* sp.[b]	Uncultured *Sebacina* clone JQ420971	**99%(499/503)**	Plant species *Vaccinium myrtillus*
OTU 41	2	2	Ascomycota	Dothideales	Dothioraceae	Dothioraceae sp.	Uncultured fungus clone KF274441	93%(412/443)	*Picea abies* stumps in Finland
							Aureobasidium sp. KJ690089	91%(402/442)	Ectomycorrhize of *Eucalyptus camaldulensis*
OTU 42	2	705	Ascomycota	Xylariales	Amphisphaeriaceae	Amphisphaeriaceae sp.	Uncultured fungus clone KC966357	**99%(518/519)**	Arctic soil in Canada (73.22N; 119.56W)
							Discosia pseudoartocreas KF777161	93%(415/448)	Plant leaves (*Tilia* sp.) in Austria (Innsbruck)
OTU 43	1	9	Ascomycota	Pleosporales	Leptosphaeriaceae	Leptosphaeriaceae sp.	Uncultured fungus clone KF742590	89%(385/432)	Imprted asymptomatic live woody plant (Berberis) material in

OTU ID	Freq.	Reads	phylum	Order	Family	Identification	Close GenBank match / Accession	Similarity	Originally reported habitat
									USA
							Leptosphaeria contecta AF181702	92%(277/300)	Unreported
OTU 44	5	26	Ascomycota	Capnodiales	Cladosporiaceae	*Rachicladosporium monterosium*[a]	Uncultured fungus clone KC966122	**97%(511/526)**	Arctic soil in USA (69.67N;148.72W)
							Rachicladosporium monterosium KF309940	**97% (403/416)**	Unreported
OTU 45	2	92	Ascomycota	Capnodiales	Teratosphaeriaceae	Teratosphaeriaceae	*Teratospaeria alistairi* JX556227	93%(358/385)	Leaves (*Protea repens*) in South Africa (Hermanus)
OTU 46	1	2	Basidiomycota	Thelephorales	Thelephoraceae	*Tomentella* sp.[b]	*Tomentella* sp. JQ711817	**98%(450/461)**	Ectomycorrhiza (*Pinus contorta*) in Canada (55.45N; 123.20W)
OTU 47	1	13	Ascomycota	Diaporthales	Gnomoniaceae	*Apiognomonia cf. errabbunda*[a]	*Apiognomonia* cf. *errabbunda* FN548166	**99%(531/536)**	Living surface-sterilised leaves of *Fagus sylvatica* in Switzerland: Hofstetten near Basle, Swiss Canopy Crane
OTU 48	3	57	Ascomycota	Helotiales	Helotiaceae	Helotiaceae sp.	*Claussenomyces* sp. HQ533008	94%(503/533)	New Zealand
OTU 49	3	201	Basidiomycota	Cystofilobasidiales	Cystofilobasidiaceae	*Udeniomyces* sp.[b]	Uncultured *Udeniomyces* clone KC753422	**99%(521/527)**	Plant (*Triticum aestivum*) stem in sweden
OTU 50	4	71	Ascomycota	Pleosporales	Phaeosphaeriaceae	*Phaeosphaeria juncophila*[a]	Uncultured fungus clone FJ820772	**99%(501/504)**	Air sample in Germany
							Phaeosphaeria juncophila AF439488	**99%(456/458)**	Plant (*Juncus articulatus*) in Switzerland
OTU 51	1	84	Ascomycota	Xylariales	Amphisphaeriaceae	Amphisphaeriaceae sp.	Uncultured fungus clone KC966357	**97%(392/405)**	Arctic soil in Canada (73.22N; 119.56W)
							Discosia pseudoartocreas KF777161	92%(396/432)	Plant leaves (*Tilia* sp.) in Austria (Innsbruck)
OTU 52	1	4	Ascomycota	Pleosporales	Lophiostomataceae	*Lophiostoma* sp.[b]	Uncultured soil fungus clone DQ420814	**98%(495/505)**	Soil in USA (Cedar Creek, Minnesota)
							Lophiostoma arundinis AJ496633	96%(484/503)	Plant
OTU 53	2	47	Basidiomycota	Leucosporidiales	Leucosporidiaceae	*Leucosporidiella muscorum*[a]	*Leucosporidiella muscorum* FR717869	**99%(488/491)**	Plant (*Paxillus involutus*) in Germany (51.5N; 11.94E)
OTU 54	3	1627	Ascomycota	Helotiales	Unassigned	*Leptodontidium* sp.[b]	Uncultured soil fungus clone EU490132	**97%(504/522)**	Savanna soil under mesquite (*Prosopis glandulosa*) in USA (33.85 N; 99.44 W)
							Leptodontidium orchidicola KF646097	96%(501/522)	Plant root of *Rosa rugosa* in Lithuania
OTU 55	1	2	Ascomycota	Capnodiales	Cladosporiaceae	Cladosporiaceae sp.	Uncultured fungus clone KC965721	95%(483/511)	Arctic soil in Canada (78.78N;103.55W)
							Rachicladosporium pini JF951145	91%(490/541)	Plant neeles (*Pinus monophylla*) in Netherland
OTU 56	1	7	Ascomycota	Capnodiales	Unassigned	*Capnocheirides* sp.[b]	*Capnocheirides* sp. FR871188	97%(428/442)	Leaf of *Rhododendron*

OTU ID	Freq.	Reads	phylum	Order	Family	Identification	Close GenBank match / Accession	Similarity	Originally reported habitat
OTU 57	1	350	Ascomycota	Pleosporales	Pleosporaceae	Pleosporaceae sp.	Uncultured mycorrhizal fungus clone AY929129	94%(490/519)	Root of *Stipa hymenoides* in semi-arid grassland (USA:Virginia Park, Canyolands National Part, UT)
							Dendryphion penicillatum JN578618	93%(443/477)	Spain
OTU 58	4	62	Ascomycota	Helotiales	Unassigned	*Tetracladium* sp.[b]	Uncultured *Tetracladium* clone KC694156	**98%(519/531)**	Roots of *Triticum aestivum* in Sweden
OTU 59	2	3	Ascomycota	Unassigned	Unassigned	Ascomycota sp.	Uncultured ascomycete clone AM902002	86%(469/544)	House dust in Finland
							Rhinocladiella sp. EU139138	86% (469/543)	Lichen (*Umbilicaria vellea*) in China (Hebei)
OTU 60	4	97	Ascomycota	Pleosporales	Pleosporineae	*Leptosphaeria* sp.[b]	Uncultured *Leptosphaeria* clone EU852362	**99%(516/519)**	Plant (*Fraxinus excelsior*) in Sweden
OTU 61	2	11	Basidiomycota	Pucciniales	Melampsoraceae	*Melampsora epitea*[a]	*Melampsora epitea* GQ479257	**97%(221/228)**	Willow in Canada (Nunavut, Keewatin District, Chesterfield Inlet)
OTU 62	1	8	Basidiomycota	Unassigned	Unassigned	Basidiomycota sp.	Uncultured fungus clone KF274456	87%(375/431)	*Picea abies* stumps in Finland
							Uncultured Basidiomycota clone GU328523	88%(355/405)	Oe layer in a fortested landscape
OTU 63	1	11	Ascomycota	Helotiales	Dermateaceae	*Helgardia* sp.[b]	Uncultured ascomycete clone AM901839	**99%(509/512)**	House dust in Finland
							Helgardia sp. HQ533816	**99%(459/462)**	Antarctic lake
OTU 64	4	13	Basidiomycota	Tremellales	Unassigned	*Cryptococcus tephrensis*[a]	*Cryptococcus tephrensis* DQ000318	**98%(501/511)**	Plant leaves
OTU 65	2	812	Ascomycota	Xylariales	Amphisphaeriaceae	Amphisphaeriaceae sp.	Uncultured fungus clone KC966357	**97%(400/413)**	Arctic soil in Canada (73.22N; 119.56W)
							Discosia pseudoartocreas KF777161	90%(395/441)	Plant leaves (*Tilia* sp.) in Austria (Innsbruck)
OTU 66	3	46	Ascomycota	Helotiales	Unassigned	*Cadophora luteo-olivacea*[a]	*Cadophora luteo-olivacea* HM116747	**99%(530/534)**	Plant (*Vitis vinifera*) in New Zealand
OTU 67	3	14	Basidiomycota	Tremellales	Unassigned	*Cryptococcus* sp.[b]	Uncultured fungus clone KC966331	**99%(481/486)**	Arctic soil in Canada (78.78N; 103.55W)
							Cryptococcus sp. KC455886	**99% (426/428)**	Snow with surperficial sediment in Italy (Helbronner peak, Mont Blanc, Italian Alps)
OTU 68	2	2	Ascomycota	Helotiales	Unassigned	Helotiales sp.	Ascomycota sp. FM207647	91%(436/480)	Surface sterilised roots of Deschampsia antarctica in Antarctica (South Orkney Islands, Shingle Cove) (60.39S, 45.33W)
							Varicosporium delicatum KF730848	94%(354/377)	Unreported

OTU ID	Freq.	Reads	phylum	Order	Family	Identification	Close GenBank match / Accession	Similarity	Originally reported habitat
OTU 69	1	11	Ascomycota	Hypocreales	Unassigned	*Acremonium rutilum*[a]	Uncultured fungus clone KC965330	**99%(445/448)**	Arctic soil in USA (70.31N; 147.99W)
							Acremonium rutilum NR077124	**97%(422/435)**	Moist greenhouse wall in Germany
OTU 70	1	17	Ascomycota	Unassigned	Unassigned	Ascomycota sp.	Uncultured fungus clone KF274173	91%(361/398)	*Picea abies* stumps in Finland
							Uncultured ascomycete clone AM901896	86%(362/422)	House dust in Finland
OTU 71	1	5	Ascomycota	Unassigned	Unassigned	Ascomycota sp.	Pleosporales sp. HQ631002	82%(356/432)	Unreported
OTU 72	5	6	Unassigned	Unassigned	Unassigned	Fungus sp.	Uncultured fungus clone KF742601	**99%(345/347)**	Imported asymptomatic live woody plant material (Prunus) in USA
OTU 73	3	46	Ascomycota	Chaetothyriales	Herpotrichiellaceae	*Rhinocladiella* sp.[b]	Uncultured fungus clone KC965227	**98%(558/570)**	Arctic soil in Canada (76.23N; 119.30W)
							Rhinocladiella sp. FJ948175	**97%(507/521)**	Rock-inhatbiting fungus from Aketao (Xinjiang, Chiina)
OTU 74	9	1048	Ascomycota	Pleosporales	Unassigned	Pleosporales sp.	Uncultured fungus clone GU817184	**98%(504/513)**	Root system (*Bistorta vivipara*) in Norway (Svalbard Midtre Lovenbreen) (78.90555N; 12.08450E)
							Pleosporales sp. AB751503	**98% (483/493)**	*Salix arctica* leaf and stem litter on deglaciated moraines in high-arctic Canada
OTU 75	1	6	Basidiomycota	Tremellales	Unassigned	Tremellales sp.	Uncultured Basidiomycota clone GU328576	91%(474/521)	Oe layer in forest
							Cryptococcus skinneri NR073211	91% (439/485)	Unreported
OTU 76	4	1076	Ascomycota	Pezizales	Unassigned	Pezizales sp.	Uncultured fungus clone KF296725	**97%(475/490)**	Arctic soil in Canada (76.23N; 119.30W)
							Phialea strobilina EF596821	92% (302/327)	Needle in Norway
OTU 77	1	252	Ascomycota	Helotiales	Unassigned	Helotiales sp.	Uncultured fungus clone KF274367	**97%(485/498)**	*Picea abies* stumps in Finland
							Helotiales sp. HQ608110	**97%(466/482)**	Trachymyrmex septentrionalis nest in USA (Stengl Biology Statioin, Texas)
OTU 78	2	77	Ascomycota	Pleosporales	Leptosphaeriaceae	Leptosphaeriaceae sp.	Uncultured fungus clone KC965764	95%(449/473)	Arctic soil in Canada (78.78N; 103.55)
							Leptosphaeria doliolum JF740206	93%(357/384)	Rudbeckia in Netherlands
OTU 79	2	217	Ascomycota	Helotiales	Unassigned	Helotiales sp.	Uncultued *Cadophora* clone GQ219956	91%(415/456)	Soil in Germany (Thuringia, Hainich)
OTU 80	4	225	Ascomycota	Unassigned	Unassigned	*Eleutheromyces subulatus*[a]	*Eleutheromyces subulatus* KJ710471	**99%(485/487)**	Decaying host (Russulaceae) in Canada (Alberta)

OTU ID	Freq.	Reads	phylum	Order	Family	Identification	Close GenBank match / Accession	Similarity	Originally reported habitat
OTU 81	1	18	Ascomycota	Xylariales	Amphisphaeriaceae	Amphisphaeriaceae sp.	Uncultured fungus clone KC966357	97%(388/398)	Arctic soil in Canada (73.22N; 119.56W)
							Discosia pseudoartocreas KF777161	90%(384/426)	Plant leaves (*Tilia* sp.) in Austria (Innsbruck)
OTU 82	8	3433	Ascomycota	Pleosporales	Phaeosphaeriaceae	*Mycopappus* sp.[b]	Uncultured soil fungus clone DQ420833	**98%(527/537)**	Soil in USA (Cedar Creek, Minnesota)
							Mycopappus aceris FJ839625	95%(504/533)	Fallen leaves (*Acer macrophyllum*) in Canada
OTU 83	2	47	Unassigned	Unassigned	Unassigned	Fungus sp.	Uncultured fungus clone KF800378	87%(426/492)	House dust in USA (Missouri, Kansas City)
OTU 84	1	36	Ascomycota	Xylariales	Amphisphaeriaceae	Amphisphaeriaceae sp.	Uncultured fungus clone KC966357	**97%(410/413)**	Arctic soil in Canada (73.22N; 119.56W)
							Discosia pseudoartocreas KF777161	90%(397/441)	Plant leaves (*Tilia* sp.) in Austria (Innsbruck)
OTU 85	4	1826	Ascomycota	Xylariales	Unassigned	Xylariales sp.	*Leiosphaerella lycopodina* JF440975	90%(479/533)	Unreported
OTU 86	3	297	Basidiomycota	Pucciniales	Melampsoraceae	*Melampsora epitea*[a]	*Melampsora epitea* GQ479257	**98%(216/220)**	Willow in Canada (Nunavut, Keewatin District, Chesterfield Inlet)
OTU 87	9	248	Ascomycota	Hypocreales	Nectriaceae	*Fusarium* sp.	Uncultured *Fusarium* sp. GQ280339	**99%(535/536)**	Plant roots (*Paphiopedilum armeniacum*) in South Western China
OTU 88	2	23	Basidiomycota	Erythrobasidiales	Unassigned	Erythrobasidiales sp.	Uncultured basidiomycete AM901803	95%(469/496)	House dust in Finland
							Erythrobasidium hasegawianum AB036065	94% (442/469)	Plant in Japan
OTU 89	2	388	Ascomycota	Unassigned	Unassigned	Ascomycota sp.	*Scleroconidioma sphagnicola* NR121294	93%(215/230)	Unreported
OTU 90	1	15	Ascomycota	Helotiales	Unassigned	Helotiales sp.	Uncultured fungus clone FM999599	95%(389/411)	Forest soil in USA (Ohio)
							Uncultured Helotiales clone FJ475762	92%(393/426)	*Pinus sylvestris* forest soil in Sweden
OTU 91	2	22	Ascomycota	Helotiales	Unassigned	Helotiales sp.	Uncultured fungus clone KF274394	**99%(434/437)**	*Picea abies* stumps in Finland
							Helotiales sp. HQ845751	92%(394/426)	Surface sterilized needle tissue (*Pinus monticola*) in USA (Antler Ridge Property, Missoula, MT)
OTU 92	3	15	Basidiomycota	Agaricostilbales	Agaricostilbaceae	Agaricostilbaceae sp.	*Bensingtonia chanbaiensis* AY233339	93%(402/432)	Unreported
OTU 93	4	1383	Ascomycota	Pleosporales	Phaeosphaeriaceae	Phaeosphaeriaceae sp.	Fungal sp. KF527818	96%(371/387)	Plant (*Carex bigelowii*) root in Finland
							Mycoppus aceris FJ839625	93%(362/390)	Fallen leaves (*Acer macrophyllum*) in Canada
OTU 94	2	577	Ascomycota	Pleosporales	Leptosphaeriaceae	*Leptosphaeria pedicularis*[a]	*Leptosphaeria pedicularis* JF740224	**99%(331/336)**	Plant (*Pedicularis*) Switzerland

OTU ID	Freq.	Reads	phylum	Order	Family	Identification	Close GenBank match / Accession	Similarity	Originally reported habitat
OTU 95	7	131	Ascomycota	Helotiales	Helotiaceae	Helotiaceae sp.	Uncultured fungus clone GU174277	95%(399/419)	Forest floor (*Acer saccharum*) in USA (Michigan, Baraga State Forest area) (46.8634N; 88.8843W)
							Articulospora tetracladia EU998929	92% (387/420)	Aquatic sample
OTU 96	3	10	Basidiomycota	Tremellales	Tremellaceae	*Dioszegia* sp.[b]	*Dioszegia* sp. DQ402529	**98%(409/416)**	Soils of Taylor Valley, Antarctica
OTU 97	1	160	Ascomycota	Chaetothyriales	Herpotrichiellaceae	Herpotrichiellaceae sp.	Uncultured fungus clone KC965841	**98%(521/534)**	Arctic soil in USA (69.67N; 148.72W)
							Capronia sp. EU139151	90%(454/506)	Lichen Umbilicariales in USA (Appalachian Mts.)
OTU 98	2	3	Ascomycota	Capnodiales	Teratosphaeriaceae	Teratosphaeriaceae sp.	Uncultured fungus clone KF274301	87%(433/497)	*Picea abies* stumps in Finland
							Teratosphaeria encephalarti FJ372400	91%(369/405)	Living leaces (*Encephalartos lebomboensis*) in South Africa
OTU 99	2	5	Basidiomycota	Cystofilobasidiales	Cystofilobasidiaceae	*Cryptococcus niccomabsii*[a]	Uncultured Basidiomycota clone AM901720	**99%(361/364)**	House dust in Finland
							Cryptococcus niccomabsii AY029346	**99%(323/325)**	Unreported
OTU 100	5	10	Ascomycota	Phacidiales	Phacidiaceae	Phacidiaceae sp.	Uncultured fungus clone JN032535	92%(490/533)	Moss litter from coniferous forest
							Allantophomopsis lycopodina JX981469	91%(478/524)	Above-ground tissue of *Lycopodium annotinum* in Poland
OTU 101	1	8	Ascomycota	Lecanorales	Porpidiaceae	*Biatora* sp.[b]	Uncultured fungus clone KC965546	**98%(385/391)**	Arctic soil in Canada (76.23N; 119.30W)
							Biatora tetramera AJ247561	95%(365/386)	Finland (Karelia australis)
OTU 102	1	39	Ascomycota	Unassigned	Unassigned	Ascomycota sp.	*Phaeothecoidea melaleuca* HQ599594	90%(508/567)	On leaves of *Melaleuca quinquenerva* in Australia
OTU 103	3	337	Ascomycota	Microascales	Halosphaeriaceae	*Monodictys arctica*[a]	Uncultured fungus clone GU817175	**99%(520/522)**	Root systems of *Bistorta vivipara* in Norway: Svalbard Midtre Lovenbreen (78.90555N; 12.08450E)
							Monodictys arctica EU686521	**98% (509/519)**	Roots of *Saxifraga oppositifolia* in Canadian High Arctic
OTU 104	2	2	Ascomycota	Unassigned	Unassigned	Ascomycota sp.	Uncultured fungus clone KF297187	**99%(391/395)**	Arctic soil in Canada (76.23N; 119.30W)
							Sarea difformis FJ903295	88%(346/394)	*Picea abies* stands of Latvia
OTU 105	4	403	Ascomycota	Capnodiales	Teratosphaeriaceae	Teratosphaeriaceae sp.	Uncultued ascomycete clone EU490102	92%(317/343)	Savanna soil under grasses in USA (33.85 N; 99.45 W)
							Teratosphaeria allistairii JX556227	92% (315/342)	Leaves (*Protea repens*) in South Africa (Hermanus)
OTU 106	1	3	Ascomycota	Hypocreales	Nectriaceae	Nectriaceae sp.	Uncultured fungus clone GU721600	87%(419/479)	Surface dust in USA
							Nectria berolinensis HM534893	90% (382/426)	Plant *Ribes sanguineum*

OTU ID	Freq.	Reads	phylum	Order	Family	Identification	Close GenBank match / Accession	Similarity	Originally reported habitat
OTU 107	1	14	Unassigned	Unassigned	Unassigned	Fungus sp.	Uncultured fungus clone KF296943	88%(345/394)	Arctic soil in Canada (76.23N; 119.30W)
OTU 108	1	3	Ascomycota	Unassigned	Unassigned	*Xenopolyscytalum* sp.[b]	Uncultured Leotiomycetes clone HM230882	**98%(438/449)**	Cavendishioid mycorrhiza of *Cavendishia bracteata* in Ecuador
							Xenopolyscytalum pinea HQ599581	95% (425/449)	Needles of *Pinus* sp. In Netherlands
OTU 109	2	4	Ascomycota	Capnodiales	Mycosphaerellaceae	Mycosphaerellaceae sp.	Uncultured fungus clone KC965961	96%(474/493)	Arctic soil in Canada (73.22N; 119.56W)
							Xenostigmina zilleri FJ839639	90% (419/468)	Fallen leaves in Canada
OTU 110	2	93	Ascomycota	Capnodiales	Cladosporiaceae	*Cladosporium* sp.[b]	Uncultured fungus clone KC965568	**99%(519/520)**	Arctic soil in Canada (73.22N; 119.56W)
							Cladosporium sp. EU167586	**97% (529/543)**	Leaf of *Trifolium*
OTU 111	7	1060	Ascomycota	Pleosporales	Phaeosphaeriaceae	*Mycopappus* sp.[b]	Uncultured fungus clone KF296865	**99%(500/505)**	Arctic soil in Canada (73.22N; 119.56W)
							Mycopappus aceris FJ839625	95% (507/532)	Fallen leaves (*Acer macrophyllum*) in Canada
OTU 112	6	184	Ascomycota	Helotiales	Unassigned	Helotiales sp.	Uncultured Helotiales isolate GU931728	95%(345/365)	House dust in Canada (45.2556N; 75.9204W)
OTU 113	1	5	Ascomycota	Unassigned	Unassigned	Ascomycota sp.	Uncultured fungus clone KC965228	**98%(368/376)**	Arctic soil in Canada (76.23N; 119.30W)
							Umbilicaria decussata AF096214	88% (397/449)	Unreported
OTU 114	1	3	Unassigned	Unassigned	Unassigned	Fungus sp.	Uncultured fungus clone JN904716	90%(204/226)	Phyllosphere of *Fagus sylvatica* in France
OTU 115	1	5	Ascomycota	Unassigned	Unassigned	Dothideomycetes sp.	Dothideomycetes sp. JQ759518	**99% (287/290)**	Surface sterilized photosynthetic tissue of Plant (*Cassiope tetragona*) in USA (64.5011N; 165.4064W)
OTU 116	8	146	Basidiomycota	Cystofilobasidiales	Cystofilobasidiaceae	*Mrakia* sp.[b]	Uncultured *Mrakia* isolate GU931743	**98%(313/321)**	House dust in Canada (45.2556N; 75.9204W)
OTU 117	1	9	Ascomycota	Helotiales	Unassigned	Helotiales sp.	*Pezizella discreta* JF908571	94%(475/508)	Italy
OTU 118	1	5	Ascomycota	Pleosporales	Leptosphaeriaceae	*Phoma sclerotioides*[a]	*Phoma sclerotioides* EU265670	**99%(473/478)**	Plant (*Medicago sativa*) root in USA (New Mexico)
OTU 119	2	231	Ascomycota	Helotiales	Vibrisseaceae	Vibrisseaceae sp.	Uncultured Vibrisseaceae clone FR773173	92%(338/367)	Plant *Rhododendron ferrugineum*
OTU 120	10	469	Ascomycota	Pleosporales	Unassigned	Pleosporales sp.	Uncultured fungus clone GU817159	**98%(421/430)**	Plant root (*Bistorta vivipara*) in Norway: Svalbard Midtre Lovenbreen (78.90555N; 12.08450E)
							Pleosporales sp. AB751503	94% (420/445)	Plant (*Salix arctica*) leaf and stem litter in high-arctic Canada
OTU 121	1	4	Unassinged	Unassigned	Unassigned	Fungus sp.	Uncultured fungus clone KF800411	84%(335/398)	House dust in USA (Missouri, Kansas City)

OTU ID	Freq.	Reads	phylum	Order	Family	Identification	Close GenBank match / Accession	Similarity	Originally reported habitat
OTU 122	2	566	Ascomycota	Hypocreales	Unassigned	Hypocreales sp.	Uncultured fungus clone GU817178	96%(512/536)	Plant root (*Bistorta vivipara*) in Norway: Svalbard Midtre Lovenbreen (78.90555N; 12.08450E)
							Uncultured Hypocreales clone FJ554224	88% (471/536)	Forest soil in from the long-term soil productivity site Skulow Lake
OTU 123	5	143	Ascomycota	Unassigned	Unassigned	*Articulospora* sp.[b]	Uncultured fungus clone GU174277	**97%(501/518)**	Forest floor (*Acer saccharum*) in USA (Michigan, Baraga State Forest area) (46.8634N; 88.8843W)
							Articulospora tetracladia EU998918	96% (496/519)	Aquatic sample
OTU 124	1	85	Ascomycota	Unassigned	Unassigned	Ascomycota sp.	Uncultured fungus clone FN610978	89%(425/480)	Sandy clayey soil from a Fagus sylvatica forest stand
							Tothia fuscella JF927786	86%(418/487)	Plant (*Teucrium chamaedrys*)
OTU 125	7	161	Basidiomycota	Tremellales	Unassigned	*Cryptococcus* sp. [b]	Uncultured *Cryptococcus* clone KC753413	**97%(360/373)**	Plant stem (*Triticum aestivum*) in Sweden
OTU 126	2	2	Ascomycota	Venturiales	Venturiaceae	Venturiaceae sp.	*Tothia fuscella* JF927786	93%(457/494)	Unreported
OTU 127	2	11	Ascomycota	Venturlales	Unassigned	Ascomycota sp.	Uncultured fungus clone AM260882	89%(310/350)	Peat in United Kingdom (57.36N; 2.90W)
							Venturia tremulae EU035475	88%(304/347)	Plant (*Populus tremula*) in Italy
OTU 128	7	705	Ascomycota	Capnodiales	Unassigned	Capnodiales sp.	Uncultured fungus clone JQ247386	95%(483/523)	Soil from transition shrubland (32.41N; 116.68W)
							Penidiella ellipsoidea JF499843	92%(483/523)	Leaf bracts (*Phaenocoma prolifera*) in South Africa
OTU 129	3	27	Basidiomycota	Tremellales	Unassigned	*Cryptococcus victoriae*[a]	*Cryptococcus victoriae* JX188144	**99%(331/334)**	Plant (*Vitis vinifera*) in USA (Central Washington State)
OTU 130	1	9	Basidiomycota	Tremellales	Unassigned	Tremellales sp.	Uncultured fungus clone KC965860	**98%(468/479)**	Arctic soil in Canada (76.23N; 119.30W)
							Cryptococcus dimennae HG008764	86%(381/444)	Air sample in France (Montignac, Lascaux Cave)
OTU 131	2	276	Ascomycota	Venturiales	Venturiaceae	*Venturia* sp.[b]	*Venturia minuta* EU035464	96%(365/379)	Plant (*Salix nigricans*) in Switzerland
OTU 132	3	102	Basidiomycota	Pucciniales	Melampsoraceae	*Melampsora epitea*[a]	*Melampsora epitea* GQ479257	**98%(228/233)**	Willow in Canada (Nunavut, Keewatin District, Chesterfield Inlet)
OTU 133	1	44	Basidiomycota	Sebacinales	Sebacinaceae	Sebacinaceae sp.	Uncultured fungus clone KF296861	**99%(493/498)**	Arctic soil in Canada (76.23N; 119.30W)
							Uncultured *Sebacina* clone JQ420971	92% (475/517)	Plant species *Vaccinium myrtillus*
OTU 134	3	1213	Ascomycota	Unassigned	Unassigned	Dothideomycetes sp.	Uncultured fungus clone KF800576	90%(510/569)	Indoor air in USA (Missouri, Kansas City)

OTU ID	Freq.	Reads	phylum	Order	Family	Identification	Close GenBank match / Accession	Similarity	Originally reported habitat
							Dothideomycetes sp. JQ759619	92% (456/498)	Surface sterilized photosynthetic tissue of Plant (*Cassiope tetragona*) in USA (64.5011N; 165.4064W)
OTU 135	1	11	Basidiomycota	Tremellales	Unassigned	Tremellales sp.	Uncultured Basidiomycota clone GU328576	95%(268/281)	Oe layer in forest
							Cryptococcus sp. HQ890370	94% (239/255)	Plant leaves in China
OTU 136	1	6	Ascomycota	Saccharomycetales	Metschnikowiaceae	*Metschnikowia* sp.[b]	*Metschnikowia bicuspidata* EF643581	95%(377/398)	Unreported
OTU 137	9	1250	Basidiomycota	Cystofilobasidiales	Cystofilobasidiaceae	*Mrakia* sp.[b]	Uncultured *Mrakia* isolate GU931743	**99%(483/490)**	House dust in Canada (45.2556N; 75.9204W)
OTU 138	3	139	Basidiomycota	Pucciniales	Melampsoraceae	*Melampsora epitea*[a]	*Melampsora epitea* GQ479257	**100%(201/201)**	Willow in Canada (Nunavut, Keewatin District, Chesterfield Inlet)
OTU 139	2	69	Ascomycota	Venturiales	Venturiaceae	*Venturia atriseda*[a]	*Venturia atriseda* EU035449	**98%(556/565)**	Plant (*Gentiana lutea*) in Switzerland
OTU 140	1	7	Ascomycota	Unassigned	Unassigned	Pezizomycotina sp.	Uncultured fungus clone KF274441	92%(239/261)	Wood stump (*Picea abies*) in Finland
							Uncultured Pezizomycotina clone FJ553309	89% (232/260)	Forest soil from the long-term soil productivity site Skulow Lake in Canada
OTU 141	5	24	Ascomycota	Thelebolales	Thelebolaceae	Thelebolaceae sp.	Uncultured Thelebolaceae clone FM178231	**99%(540/543)**	Malthouse waster water
OTU 142	5	636	Unassigned	Unassigned	Unassigned	*Phaeosphaeria* sp.[b]	Uncultued ascomycete clone AM901822	96%(421/439)	House dust in Finland
							Phaeosphaeria triglochinicola AF439507	96% (364/379)	Plant (*Triglochin palustris*) in Switzerland
OTU 143	1	3	Ascomycota	Unassigned	Unassigned	Pezizomycotina sp.	Uncultured fungus clone KF274441	94%(459/487)	Wood stump (*Picea abies*) in Finland
							Uncultured Pezizomycotina clone FJ553309	89% (466/523)	Forest soil from the long-term soil productivity site Skulow Lake
OTU 144	1	24	Unassigned	Unassigned	Unassigned	Fungus sp.	Uncultured fungus clone EF521234	89%(283/319)	Mesh bags buried in a phosphorus-poor spruce forest
OTU 145	1	306	Ascomycota	Unassigned	Unassigned	*Scleroconidioma* sp.[b]	*Scleroconidioma sphagnicola* NR121294	95%(541/569)	Unreported
OTU 146	4	9	Ascomycota	Capnodiales	Teratosphaeriaceae	Teratosphaeriaceae sp.	Uncultured fungus clone KF297284	**99%(502/509)**	Arctic soil in Canada (Banks Island) (73.22N; 119.56W)
							Uncultured *Devriesia* clone JF519086	90% (459/511)	*Fagus sylvatica* lateral root in Austria (Klausenleopoldsdorf)
OTU 147	2	38	Ascomycota	Xylariales	Xylariaceae	Xylariaceae sp.	Uncultured Xylariaceae isolate EU326164	**98%(429/437)**	Ectomycorrhizal root tip of *Salix herbacea* on a glacier forefront in the Austrian Alps

OTU ID	Freq.	Reads	phylum	Order	Family	Identification	Close GenBank match / Accession	Similarity	Originally reported habitat
OTU 148	2	5	Ascomycota	Capnodiales	Unassigned	*Elasticomyces elasticus*[a]	*Elasticomyces elasticus* FJ415476	**98%(448/457)**	Lichen in Antarctica
OTU 149	3	28	Basidiomycota	Tremellales	Unassigned	*Dioszegia fristingensis*[a]	*Dioszegia fristingensis* JQ768931	**99%(479/480)**	Glacier surface snow in China (Tibet plateau)
OTU 150	2	20	Ascomycota	Capnodiales	Cladosporiaceae	*Rachicladosporium monterosium*[a]	Uncultured fungus clone KC966347	**97%(495/511)**	Arctic soil in Canada (76.23N; 119.30W)
							Rachicladosporium monterosium KF309940	**98%(464/472)**	Unreported
OTU 151	4	10	Ascomycota	Microascales	Halosphaeriaceae	*Monodictys* sp.[b]	*Monodictys arcitica* EU686521	96%(419/435)	Roots of *Saxifraga oppositifolia* in Canadian High Arctic
OTU 152	1	30	Ascomycota	Helotiales	Unassigned	Helotiales sp.	Uncultured fungus clone KC965667	**98%(461/470)**	Arctic soil in USA (69.15N; 148.85W)
							Uncultured Helotiales clone FJ827194	96%(326/341)	Ectomycorrhiza of *Potentilla* sp. on an alpine-meadow in southwestern China
OTU 153	1	5	Ascomycota	Chaetothyriales	Trichomeriaceae	Trichomeriaceae sp.	Uncultured soil fungus clone DQ421063	**99%(559/568)**	Soil in USA (Cedar Creek, Minnesota)
							Knufia petricola KC978734	94%(549/582)	Unreported
OTU 154	1	12	Ascomycota	Helotiales	Unassigned	Helotiales sp.	Uncultured fungus clone KF742558	**97%(349/358)**	Imported asymptomatic live woody plant material (*Acer*) in USA
							Uncultured Helotiales clone KF498574	87%(415/478)	Extracted from fiine roots (Fagus sylvatica) in Germany (Beech forest near Tuttlingen, Swabian)
OTU 155	2	14	Basidiomycota	Unassigned	Unassigned	Basidiomycota sp.	Uncultured basidiomycete AM901997	**98%(367/375)**	House dust in Finland
OTU 156	1	7	Ascomycota	Helotiales	Unassigned	*Varicosporium elodeae*[a]	Uncultured fungus clone KF617967	90%(346/386)	Picea mariana forest soil organic horizon in USA (Alaska, Bonaza Creek LTER) (63.9062N; 145.3712W)
							Varicosporium elodeae JN995640	**97%(257/264)**	Svalbard
OTU 157	3	2383	Ascomycota	Hypocreales	Unassigned	Hypocreales sp.	Uncultured fungus clone GU817178	95%(406/427)	Root system of *Bistorta vivipara* in Norway (Svalbard Midtre Lovenbreen) (78.90N; 12.08E)
							Myrothecium sp. KF723006	90%(381/423)	Aerated munifipal sewage sludge in India (Koyambedu, Chennai)
OTU 158	1	3	Ascomycota	Chaetothyriales	Herpotrichiellaceae	Herpotrichiellaceae sp.	Uncultured ascomycete clone AM901730	91%(386/424)	House dust in Finland
							Coniosporium sp. AJ971446	90%(381/423)	Marble monument in Turkey (Mediterranean)

OTU ID	Freq.	Reads	phylum	Order	Family	Identification	Close GenBank match / Accession	Similarity	Originally reported habitat
OTU 159	6	471	Ascomycota	Capnodiales	Mycosphaerellaceae	Mycosphaerellaceae sp.	Uncultured soil fungus clone DQ420833	**97%(514/530)**	Soil in USA (Cedar Creek, Minnesota)
							Xenostigmina zilleri FJ839639	94% (503/535)	Fallen leaves of *Acer macrophyllum* in Canada
OTU 160	1	12	Ascomycota	Helotiales	Helotiaceae	Helotiaceae sp.	*Gremmeniella laricina* U72262	94%(343/363)	From *Larix decidua* in Switzerland
OTU 161	2	25	Basidiomycota	Pucciniales	Melampsoraceae	*Melampsora* sp.[b]	Uncultured *Melampsora* clone JN646138	95%(328/344)	Small discs of infected leaf material of Saflix arbuscula in United Kingdom (Scotland, Beinn Heasgarnich)
OTU 162	2	12	Ascomycota	Unassigned	Unassigned	Ascomycota sp.	Uncultured fungus clone KF274173	89%(446/500)	*Picea abies* stumps in Finland
							Uncultured Helotiales clone FJ827194	85%(382/448)	Ectomycorrhiza of *Potentilla* sp. on an alpine-meadow in southwestern China
OTU 163	2	115	Ascomycota	Unassigned	Unassigned	Ascomycota sp.	Uncultured fungus clone KF296725	**99%(485/491)**	Arctic soil in Canada (76.23N; 119.30W)
							Hyaloscyphaceae sp. GU393951	84%(443/527)	Scots pine needles (*Pinus sylvestris*) in Sweden
OTU 164	5	225	Ascomycota	Helotiales	Unassigned	Helotiales sp.	*Leptodontidium orchidicola* KF646097	94%(461/490)	Plant root of *Rosa rugosa* in Lithuania
OTU 165	1	19	Ascomycota	Unassigned	Unassigned	Fungus sp.	Uncultured fungus clone KC966118	**99%(510/512)**	Arctic soil in USA (69.67N;148.72W)
OTU 166	7	1696	Ascomycota	Helotiales	Helotiaceae	*Articulospora* sp.[b]	Uncultured fungus clone KC966259	**99%(482/489)**	Arctic soil in Canada (76.23N;119.30W)
							Articulospora tetracladia EU998918	96%(482/504)	Aquatic sample
OTU 167	1	7	Basidiomycota	Naohideales	Unassigned	Naohideales sp.	*Naohidea sebaceae* NR121324	89%(429/482)	Taiwan
OTU 168	7	189	Ascomycota	Pleosporales	Melanommataceae	Melanommataceae sp.	Fungal sp. KF527818	95%(444/467)	Plant (*Carex bigelowii*) root in Finland
							Herpotrichia juniperi JX981496	94%(405/430)	Plant in Poland
OTU 169	1	276	Chytridiomycota	Chytridiales	Unassigned	Chytridiales sp.	*Synchytrium cupulatum* KF160867	89%(221/247)	Plant (*Dryas octopetala*) in Finland
OTU 170	3	53	Ascomycota	Capnodiales	Unassigned	*Penidiella* sp.[b]	*Penidiella ellipsoidea* JF499843	95%(455/480)	Leaf bracts (*Phaenocoma prolifera*) in South Africa
OTU 171	3	99	Ascomycota	Helotiales	Helotiaceae	Helotiaceae sp.	*Claussenomyces* sp. HQ533008	94%(472/503)	New Zealand
OTU 172	1	587	Ascomycota	Helotiales	Unassigned	*Tetracladium* sp.[b]	Uncultured *Tetracladium* clone KC694156	95%(510/539)	Roots of *Triticum aestivum* in Sweden
OTU 173	3	6	Ascomycota	Helotiales	Leotiaceae	*Alatospora* sp.[b]	Uncultured *Alatospora* clone JF519259	96%(381/398)	*Fagus sylvatica* lateral root in Austria (48.11N; 16.05E)
OTU 174	7	391	Unassigned	Unassigned	Unassigned	Fungus sp.	Uncultured fungus clone KF742601	95%(466/488)	Imported asymptomatic live woody plant material (Prunus) in USA

OTU ID	Freq.	Reads	phylum	Order	Family	Identification	Close GenBank match / Accession	Similarity	Originally reported habitat
OTU 175	3	10	Ascomycota	Helotiales	Unassigned	Helotiales sp.	Uncultured Helotiales clone FJ827183	91%(487/536)	Ectomycorrhiza of *Potentilla* sp. on alpine-meadow in southwestern China
OTU 176	3	347	Ascomycota	Venturiales	Venturiaceae	*Venturia minuta*[a]	*Venturia minuta* EU035464	**97%(541/556)**	Plant (*Salix nigricans*) in Switzerland
OTU 177	1	5	Ascomycota	Microascales	Halosphaeriaceae	*Monodictys* sp.[b]	Uncultured fungus clone GU817175	96%(449/468)	Root systems of *Bistorta vivipara* in Norway (Svalbard Midtre Lovenbreen) (78.90N; 12.08E)
							Monodictys arctica EU686521	96%(448/467)	Roots of *Saxifraga oppositifolia* in Canadian High Arctic
OTU 178	1	23	Basidiomycota	Unassigned	Unassigned	Basidiomycota sp.	Uncultured fungus clone EF521234	91%(419/462)	Mesh bags in a phosphorus-poor spruce forest
							Basidiomycete sp. AM084802	89%(351/396)	Boreal forests in Norway
OTU 179	5	706	Ascomycota	Helotiales	Unassigned	*Tetracladium* sp.[b]	Uncultured soil fungus clone JQ666656	**98%(519/532)**	Forest soil in northern temperate forest (China, Changbai Mountain)
							Tetracladium furcatum EU883432	96%(513/533)	Auqtic sample
OTU 180	3	221	Ascomycota	Unassigned	Unassigned	Ascomycota sp.	Uncultured fungus clone KC966025	95%(365/384)	Arctic soil in Canada (76.23N; 119.30W)
							Pseudeurotium bakeri GU934582	86%(491/547)	Root systems from mixed *Fraxinus excelsior* stands with rich soils and badly damaged by fungal attacks in Lithuania
OTU 181	4	24	Ascomycota	Xylariales	Amphisphaeriaceae	Amphisphaeriaceae sp.	Uncultured fungus clone KC965587	98%(483/495)	Arctic soil in Canada (73.22N; 119.56W)
							Seimatosporium walkeri JN871207	94% (490/523)	Plant (*Eucalyptus* sp.) in Australia
OTU 182	6	1066	Ascomycota	Helotiales	Unassigned	*Tetracladium* sp.[b]	Uncultured *Tetracladium* clone KC753432	**97%(515/530)**	Roots of *Triticum aestivum* in Sweden
OTU 183	2	496	Ascomycota	Pleosporales	Leptosphaeriaceae	*Leptosphaeria pedicularis*[a]	*Leptosphaeria pedicularis* JF740224	**98%(456/463)**	Pediculais in Switzerland
OTU 184	1	65	Ascomycota	Capnodiales	Unassigned	Capnodiales sp.	Uncultured Capnodiales clone KF922740	90%(454/504)	Apple surface in USA (Iowa)
OTU 185	4	1196	Ascomycota	Xylariales	Amphisphaeriaceae	Amphisphaeriaceae sp.	*Leiosphaerella lycopodina* JF440975	91%(375/410)	Unreported
OTU 186	9	2394	Basidiomycota	Tremellales	Unassigned	*Cryptococcus* sp.[b]	Uncultured basidiomycete clone AM901845	**97%(359/369)**	House dust in Finland
							Uncultured *Cryptococcus* clone JF495244	**97%(359/370)**	Beech litter in Austria (Lower Austria, Klausenleopoldsdorf)
OTU 187	4	314	Ascomycota	Capnodiales	Unassigned	Capnodiales sp.	Uncultured fungus clone KC965669	**99%(497/504)**	Arctic soil in USA (69.15N; 148.85W)
							Penidiella ellipsoidea JF499843	91%(455/500)	Leaf bracts (*Phaenocoma prolifera*) in South Africa

OTU ID	Freq.	Reads	phylum	Order	Family	Identification	Close GenBank match / Accession	Similarity	Originally reported habitat
OTU 188	1	2	Ascomycota	Helotiales	Sclerotiniaceae	*Sclerotinia* sp.[b]	*Sclerotinia* sp. AJ279480	**99%(526/529)**	Unreported
OTU 189	5	352	Ascomycota	Helotiales	Unassigned	Helotiales sp.	Uncultured Helotiales clone GU998549	**98%(500/508)**	Ectomycorrhiza root tip region of Betula nana in USA, Toolik Lake, AK (68.63N; 149.57W)
OTU 190	1	10	Ascomycota	Helotiales	Hyaloscyphaceae	*Mycoarthris* sp.[b]	Uncultured Helotiales isolate GU931728	96%(498/521)	House dust in Canada (45.2556N; 75.9204W)
							Uncultured *Mycoarthris* clone JF449666	96%(480/499)	Beech litter in Austria (Carinthia, Ossiach)
OTU 191	6	767	Ascomycota	Helotiales	Unassigned	*Lemonniera* sp.[b]	Uncultured fungus clone GU174277	**98%(519/529)**	Forest floor (*Acer saccharum*) in USA (46.86N; 88.88W)
							Uncultured *Lemonniera* clone JF449671	96%(490/509)	Beech litter in Austria (Carinthia, Ossiach)
OTU 192	1	8	Basidiomycota	Agaricales	Cortinariaceae	Cortinariaceae sp.	Uncultured fungus clone KC965950	93%(470/506)	Arctic soil in Canada (73.22N; 119.56W)
							Uncultured *Hebeloma* clone JQ724055	92%(486/531)	Ectomycorrhiza of *Salix fragilis* in Sweden
OTU 193	1	101	Ascomycota	Helotiales	Helotiaceae	Helotiaceae sp.	Uncultued fungus clone KC966294	94%(477/505)	Arctic soil in USA (69.15N; 148.85W)
							Claussenomyces sp. HQ533008	92%(490/530)	New Zealand
OTU 194	2	48	Ascomycota	Helotiales	Unassigned	Helotiales sp.	Uncultured Helotiales clone KC455325	**99%(487/489)**	Root system in Arctic tundra (USA, Anaktuvuk River Fire, AK)
OTU 195	2	10	Ascomycota	Helotiales	Unassigned	*Tetracladium* sp.[b]	Uncultured *Tetracladium* clone KC753432	**97%(353/364)**	Roots of *Triticum aestivum* in Sweden
OTU 196	2	37	Ascomycota	Helotiales	Leotiaceae	*Alatospora flagellata*[a]	*Alatospora flagellata* KC834041	**98%(500/509)**	Stream, Fagus sylvatica leaf, CZ
OTU 197	2	125	Ascomycota	Helotiales	Unassigned	Helotiales sp.	Uncultured fungus clone KC965311	**99%(511/517)**	Arctic soil in Canada (73.22N; 119.56W)
							Uncultured *Tetracladium* clone GU327472	94% (507/541)	Mycorrhizal seedling (*Epipactis atrorubens*) in Czech
OTU 198	2	8	Ascomycota	Verrucariales	Verrucariaceae	*Polyblastia wheldonii*[a]	Uncultured fungus clone KC966360	**100%(479/479)**	Arctic soil in Canada (73.22N; 119.56W)
							Polyblastia wheldonii EU553497	**98%(428/436)**	Sweden
OTU 199	1	17	Ascomycota	Helotiales	Unassigned	Helotiales sp.	Uncultured fungus clone KF274382	96%(464/482)	*Picea abies* stumps in Finland
							Helotiales sp. AY465452	96% (400/417)	Surface-sterilized needle (Pnius monticola) in USA (Idaho, Long Cavon)
OTU 200	2	42	Ascomycota	Chaetothyriales	Unassigned	Chaetothyriales sp.	Uncultured fungus clone KF274441	94%(458/489)	Wood stump (*Picea abies*) in Finland
							Sarcinomyces crustaceus NR121503	90%(470/528)	Unreported
OTU 201	2	333	Ascomycota	Capnodiales	Teratosphaeriaceae	Teratosphaeriaceae sp.	*Teratosphaeria encephalari* FJ372400	93%(390/420)	Living leaves (*Encephalartos lebomboensis*) in South Africa

OTU ID	Freq.	Reads	phylum	Order	Family	Identification	Close GenBank match / Accession	Similarity	Originally reported habitat
OTU 202	3	16	Basidiomycota	Trichosporonales	Trichosporonaceae	*Trichosporon loubieri*[a]	*Trichosporon loubieri* KC254110	**98%(488/498)**	Nails in Greece
OTU 203	6	201	Ascomycota	Helotiales	Unassigned	*Tetracladium* sp.[b]	Uncultured fungus clone KC965366	**99%(496/498)**	Arctic soil in Canada (76.23N; 119.30W)
							Uncultured *Tetracladium* clone KC694157	96%(500/522)	Plant (*Triticum aestivum*) roots in Sweden
OTU 204	3	14	Ascomycota	Unassigned	Unassigned	Ascomycota sp.	Uncultured fungus clone KC965324	**99%(359/360)**	Arctic soil in USA (70.31N; 147.99W)
							Uncultured Ascomycota clone FN555433	87%(342/391)	Liverwort (*Lophozia excisa*) in Antarctica (western Antarctic Peninsula, Leonie Island)
OTU 205	4	6	Ascomycota	Unassigned	Unassigned	*Mycosymbioces* sp.[b]	Uncultured fungus clone KF296894	**99%(465/468)**	Arcitc soil in Canada (78.78N; 103.55W)
							Mycosymbioces mycenaphila KF030236	96%(418/435)	Growing with Mycena sp. in USA (Oregon)
OTU 206	6	1844	Ascomycota	Pleosporales	Phaeosphaeriaceae	*Phaeosphaeria triglochinicola*[a]	Uncultured ascomycete clone AM901822	**98%(519/528)**	House dust in Finland
							Phaeosphaeria triglochinicola AF439507	**99% (463/468)**	Plant (*Triglochin palustris*) in Switzerland
OTU 207	4	3252	Ascomycota	Helotiales	Helotiaceae	Helotiaceae sp.	*Articulospora tetracladia* EU998926	91%(457/502)	Aquatic sample
OTU 208	2	20	Ascomycota	Helotiales	Unassigned	Helotiales sp.	Uncultured soil fungus clone JQ666493	98%(497/506)	Soil in a northern temperate forest (China, Changbai Mountain)
							Uncultured Helotiales clone JF748081	93%(474/510)	Ectomycorrhiza of Quercus liaotungensis in the Chinese Loess Plateau
OTU 209	1	156	Basidiomycota	Sebacinales	Sebacinaceae	Sebacinaceae sp.	Uncultured fungus clone KC966121	94%(473/505)	Arctic soil in USA (69.67N; 148.72W)
							Sebacina vermifera DQ520096	92%(489/531)	Unreported
OTU 210	2	79	Ascomycota	Unassigned	Unassigned	Ascomycota sp.	Uncultured fungus clone KF296855	94%(490/519)	Arctic soil in Canada (78.78N; 103.55W)
							Oleoguttula mirabilis KF309972	94%(443/472)	Unreported
OTU 211	1	3	Ascomycota	Unassigned	Unassigned	Fungus sp.	Uncultured fungus clone JX364434	92%(315/341)	Soil in USA
OTU 212	7	37	Ascomycota	Venturiales	Venturiaceae	*Venturia* sp.[b]	*Venturia* sp. AB916509	**99%(500/501)**	Bird feather in Norway
OTU 213	4	99	Ascomycota	Helotiales	Unassigned	*Tetracladium* sp.[b]	Uncultured ascomycete clone FJ378864	96%(511/531)	Ectomycorrhiza of *Kobresia* sp. in Eastern Himalaya
							Uncultured *Tetracladium* clone FJ803966	96% (510/532)	Root tip from trees (*Populus davidiana*) in boreal temperate forest from linner Monoglia in China

OTU ID	Freq.	Reads	phylum	Order	Family	Identification	Close GenBank match / Accession	Similarity	Originally reported habitat
OTU 214	1	12	Basidiomycota	Agaricales	Inocybaceae	Inocybaceae sp.	Uncultured fungus clone KC965838	94%(335/356)	Arctic soil in USA (69.67N; 148.72W)
							Inocybe ochroalba EU326165	93% (315/338)	Ectomycorrhizal root tip of *Salix herbacea* in Austria (46.83N; 11.02E)
OTU 215	10	1480	Basidiomycota	Tremellales	Unassigned	*Cryptococcus* sp.[b]	Uncultured *Cryptococcus* clone KC753413	**99%(354/357)**	Plant (*Triticum aestivum*) stem in Sweden
OTU 216	1	12	Basidiomycota	Pucciniales	Melampsoraceae	Melampsoraceae sp.	*Melampsora* sp. JF825971	94%(360/382)	Plant (*Salix viminalis*) in Sweden (Skane)
OTU 217	1	2	Ascomycota	Saccharomycetales	Saccharomycetaceae	*Komagataella pastoris*[a]	*Komagataella pastoris* KF468220	**99%(288/291)**	Unreported
OTU 218	1	164	Ascomycota	Helotiales	Helotiaceae	*Claussenomyces* sp.[b]	Uncultured fungus clone KC966294	**100%(500/500)**	Arctic soil in USA (69.15N; 148.85W)
							Claussenomyces sp. HQ533008	96%(502/524)	New Zealand
OTU 219	3	12	Ascomycota	Helotiales	Unassigned	Helotiales sp.	Uncultured Helotiales clone JN847454	**100%(529/529)**	Ectomycorrhizal root tips of *Tristaniopsis* spp. in New Caledonia
OTU 220	3	29	Ascomycota	Helotiales	Helotiaceae	*Hymenoscyphus caudatus*[a]	*Hymenoscyphus caudatus* KC481687	**97%(497/511)**	Plant (*Alnus* sp.) in Germany
OTU 221	3	810	Ascomycota	Helotiales	Helotiaceae	Helotiaceae sp.	Uncultured *Helicodendron* isolate GU931747	92%(445/486)	House dust in Canada (45.2556 N, 75.9204W)
OTU 222	8	266	Basidiomycota	Tremellales	Unassigned	*Cryptococcus* sp.[b]	Uncultured *Cryptococcus* clone KC753413	**98%(502/514)**	Plant (*Triticum aestivum*) stem in Sweden
OTU 223	6	4749	Ascomycota	Unassigned	Unassigned	Dothideomycetes sp.	Dothideomycetes sp. JQ759518	**100%(447/447)**	Surface sterilized photosynthetic tissue of Plant (*Cassiope tetragona*) in USA (64.5011N; 165.4064W)
							Venturia hystrioides EU035459	86% (485/563)	Unreported
OTU 224	1	27	Ascomycota	Unassigned	Unassigned	Ascomycota sp.	Uncultured fungus clone KC965795	93%(450/484)	Arctic soil in Canada (76.23N; 119.30W)
							Uncultured ascomycete clone AM901946	84%(342/406)	House dust in Finland
OTU 225	1	14	Ascomycota	Unassigned	Unassigned	Ascomycota sp.	*Sympodiella acicola* EU449953	86%(324/378)	Litter needles (*Pinus sylvestris*) in Czech
OTU 226	1	3	Ascomycota	Helotiales	Unassigned	Helotiales sp.	Uncultured *Tetracladium* clone KC753432	92%(458/496)	Roots of *Triticum aestivum* in Sweden
OTU 227	4	44	Ascomycota	Saccharomycetales	Saccharomycetaceae	*Pichia pastoris*[a]	*Pichia pastoris* FR839631	**99%(376/379)**	Unreported
OTU 228	6	776	Ascomycota	Helotiales	Helotiaceae	Helotiaceae sp.	Uncultured fungus clone KF274177	96%(395/412)	*Picea abies* stumps in Finland
							Articulospora tetracladia EU998926	94% (390/413)	Aquatic sample
OTU 229	2	62	Ascomycota	Helotiales	Unassigned	*Tetracladium furcatum*[a]	*Tetracladium furcatum* EU883432	**97%(519/533)**	Aquatic sample

OTU ID	Freq.	Reads	phylum	Order	Family	Identification	Close GenBank match / Accession	Similarity	Originally reported habitat
OTU 230	6	45	Ascomycota	Saccharomycetales	Saccharomycetaceae	*Pichia pastoris*[a]	*Pichia pastoris* FR839630	**98%(386/394)**	Unreported
OTU 231	5	235	Ascomycota	Helotiales	Helotiaceae	*Tricladium* sp.[b]	Uncultured fungus clone KC965406	**99%(507/509)**	Arctic soil in Canada (76.23N; 119.30W)
							Tricladium angulatum AY204609	96%(485/506)	Aquatic sample
OTU 232	8	339	Ascomycota	Helotiales	Unassigned	*Tetracladium furcatum*[a]	*Tetracladium furcatum* EU883432	**98%(482/491)**	Aquatic sample
OTU 233	3	91	Ascomycota	Unassigned	Unassigned	Ascomycota sp.	Ascomycete sp. AM084848	95%(361/380)	Air sample in boreal forest (Norway)
OTU 234	3	46	Ascomycota	Helotiales	Unassigned	Helotiales sp.	Uncultured fungus clone KC965311	92%(492/533)	Arctic soil in Canada (73.22N; 119.56W)
							Uncultured *Tetracladium* clone FJ803966	90%(500/556)	Root tip from trees in boreal temperate forest (China, inner Mongolia)
OTU 235	1	153	Ascomycota	Xylariales	Amphisphaeriaceae	Amphisphaeriaceae sp.	Uncultured Amphisphaeriaceae clone FR773218	**98%(462/471)**	Unreported
OTU 236	2	15	Basidiomycota	Tremellales	Unassigned	Tremellales sp.	Uncultured fungus clone KF800558	91%(475/520)	Indoor air in USA (Missouri, Kansas City)
							Cryptococcus dimennae HG008764	86% (367/425)	Air sample in France (Montignac, Lascaux Cave)
OTU 237	3	408	Basidiomycota	Sebacinales	Sebacinaceae	Sebacinaceae sp.	Uncultured fungus clone KC966121	95%(452/476)	Arctic soil in USA (69.67N; 148.72W)
							Uncultured *Sebacina* clone HQ180275	92%(464/504)	Roots of herbaceous plants (*Trifolicum pratense*)
OTU 238	2	18	Ascomycota	Pleosporales	Pleosporineae	*Leptosphaeria* sp.[b]	Uncultured *Leptosphaeria* clone EU852362	**97%(390/402)**	Plant (*Fraxinus excelsior*) in Sweden
OTU 239	2	48	Ascomycota	Helotiales	Unassigned	Helotiales sp.	Uncultured fungus clone KC966120	**99%(427/429)**	Arctic soil in USA (69.67N; 14872W)
							Cadophora malorum GU067760	90%(407/453)	Stump (*Picea abies*) in Finland
OTU 240	2	155	Ascomycota	Pleosporales	Unassigned	*Phoma herbarum*[a]	Unculured *Epicoccum* clone FN539073	90%(478/533)	Beech sapwood in United Kingdom (53.63N; 7.69W)
							Phoma herbarum FN868459	**97% (382/394)**	Spanish stands of *Pinus halepensis*
OTU 241	3	11	Ascomycota	Unassigned	Unassigned	Ascomycota sp.	Uncultured fungus clone KF296938	**99%(452/453)**	Arctic soil in Canada (76.23N; 119.30W)
							Umbilicaria hyperborea AF096216	86%(416/485)	Unreported
OTU 242	1	5	Ascomycota	Helotiales	Unassigned	Helotiales sp.	*Lambertella* sp. AB705255	94%(378/402)	Unreported
OTU 243	1	38	Ascomycota	Chaetothyriales	Herpotrichiellaceae	*Rhinocladiella* sp.[b]	Uncultured fungus clone KC965227	**97%(538/557)**	Arctic soil in Canada (76.23N; 119.30W)
							Rhinocladiella sp. FJ948175	**97%(499/512)**	Rock-inhatbiting fungus from Aketao (Xinjiang, Chiina)
OTU 244	2	1045	Ascomycota	Venturiales	Venturiaceae	*Venturia alpina*[a]	*Venturia alpina* EU035446	**98%(561/571)**	Plant (*Arctostaphylos alpina*) in Switzerland

OTU ID	Freq.	Reads	phylum	Order	Family	Identification	Close GenBank match / Accession	Similarity	Originally reported habitat
OTU 245	6	779	Ascomycota	Helotiales	Hyaloscyphaceae	Hyaloscyphaceae sp.	Uncultured Helotiales isolate GU931728	96%(474/494)	House dust in Canada (45.2556N; 75.9204W)
OTU 246	2	3	Ascomycota	Capnodiales	Teratosphaeriaceae	Teratosphaeriaceae sp.	*Teratosphaeria parva* EU707877	92%(302/346)	Plant (*Protea nitida*) leaf in South Africa
OTU 247	4	3776	Basidiomycota	Pucciniales	Melampsoraceae	*Melampsora* sp.[b]	Uncultured *Melampsora* clone JN646154	**99%(492/497)**	Plant (*Salix reticulata*) in United Kingdom (Scotland)
OTU 248	1	22	Ascomycota	Chaetothyriales	Herpotrichiellaceae	Herpotrichiellaceae sp.	Uncultured endophytic fungus clone KF060234	90%(496/552)	Plant (*Decalepis arayalpathra*) root in India
							Phaeomoniella sp. JN225891	90% (446/494)	Plant (*Nothofagus menziesii*) leaves in New Zealand
OTU 249	3	678	Ascomycota	Pleosporales	Unassigned	*Phoma herbarum*[a]	*Phoma herbarum* FN868459	**99%(518/522)**	Spanish stands of *Pinus halepensis*
OTU 250	1	6	Basidiomycota	Pucciniales	Melampsoraceae	*Melampsora* sp.[b]	Uncultured *Melampsora* clone KC883611	**98%(394/404)**	uredospores on infected leaves (*Salix caprea* x *purpurea*)

[a]For sequence identities ≥ 97%, the genus and species were accepted.

[b]For sequence identities between 95% and 97%, only the genus was accepted.
