# Supplementary material for: Endophytic Fungal Communities Associated with Vascular Plants in the High Arctic Zone Are Highly Diverse and Host-Plant Specific
Source: PLoS One. 2015 Jun 12;10(6):e0130051. doi: 10.1371/journal.pone.0130051 (PMC4466372; doi:10.1371/journal.pone.0130051)
Supplement: S2 Table — (PDF) [file pone.0130051.s002.pdf]

S2 Table. Distribution of the 250 OTUs found in the 12 plant samples, including their number of reads.

	Ct-1	Ct-2	Ct-3	Sc-1	Sc-2	Sc-3	Sc-4	So-1	So-2	So-3	Sa-1	Sa-2
OTU1	0	1	1	342	122	10	24	55	213	4	0	7
OTU2	5216	0	8	0	0	0	0	0	0	1	0	1
OTU3	0	3	0	0	0	0	0	0	2	0	0	0
OTU4	0	0	0	0	0	0	0	0	0	0	4	1
OTU5	0	0	0	0	0	0	0	0	0	0	16	0
OTU6	0	0	0	14	0	2	27	9	387	30	0	0
OTU7	0	11	0	0	0	0	0	0	0	0	0	0
OTU8	0	3	0	0	0	0	0	0	0	0	1	0
OTU9	45	1	96	0	0	0	0	0	0	0	0	0
OTU10	0	70	0	0	0	0	0	0	0	0	0	0
OTU11	27	1	1	0	0	0	0	0	1	0	0	0
OTU12	0	0	0	0	1	0	0	0	1	0	0	0
OTU13	0	0	2	0	0	0	0	0	0	0	0	0
OTU14	0	0	4	0	0	0	0	0	0	0	0	0
OTU15	0	0	0	94	0	0	0	58	0	0	0	0
OTU16	0	17	0	0	0	0	0	0	0	0	0	0
OTU17	0	0	0	0	0	0	0	0	3	0	0	0
OTU18	0	0	0	26	15	5	24	18	156	0	0	3
OTU19	2	54	0	0	0	0	0	0	0	0	1	0
OTU20	0	0	0	0	2	0	0	0	0	0	127	0
OTU21	0	0	0	0	4	0	0	0	0	0	0	0
OTU22	2	2	0	1	0	0	1	18	50	550	0	0
OTU23	3	0	0	0	0	0	0	0	0	0	0	0
OTU24	0	0	0	5	2	3	0	0	0	0	0	0
OTU25	60	0	1	0	0	0	0	0	0	0	0	0
OTU26	90	435	7	0	0	0	0	0	1	0	1	0
OTU27	0	0	0	36	0	0	1	0	0	0	0	0
OTU28	3	0	0	1366	67	33	144	39	10	3	1	302
OTU29	0	0	0	0	0	0	7	0	0	0	0	0
OTU30	0	0	0	7	14	234	1	21	0	0	0	0
OTU31	2	0	0	200	10	2	3	1	1	0	0	39
OTU32	0	0	0	0	1	0	16	73	1	2	0	11
OTU33	99	10	346	0	0	0	0	0	0	0	0	0
OTU34	606	1	22	0	0	0	0	0	0	0	0	0
OTU35	0	0	0	0	0	0	12	1	0	0	0	0
OTU36	0	9	0	0	10	0	1	6	175	0	0	0
OTU37	0	2	0	0	0	0	0	0	0	40	0	0
OTU38	0	0	0	0	434	0	1	0	0	0	0	0
OTU39	4	3	0	19	0	6	26	144	0	679	0	0
OTU40	0	0	6	336	0	0	0	0	0	0	0	0

OTU41	0	1	0	1	0	0	0	0	0	0	0	0
OTU42	0	704	0	0	0	0	0	0	0	0	1	0
OTU43	0	0	0	0	0	0	0	9	0	0	0	0
OTU44	1	0	1	0	4	7	0	13	0	0	0	0
OTU45	90	0	2	0	0	0	0	0	0	0	0	0
OTU46	0	0	0	0	0	0	0	0	2	0	0	0
OTU47	13	0	0	0	0	0	0	0	0	0	0	0
OTU48	0	20	0	4	0	0	0	0	33	0	0	0
OTU49	0	0	0	1	0	0	0	199	0	1	0	0
OTU50	0	0	0	0	0	1	21	48	0	0	1	0
OTU51	0	84	0	0	0	0	0	0	0	0	0	0
OTU52	0	0	0	0	0	0	0	4	0	0	0	0
OTU53	0	0	0	10	0	0	0	0	0	0	0	37
OTU54	0	0	0	0	0	0	1611	12	0	4	0	0
OTU55	0	0	0	0	0	0	2	0	0	0	0	0
OTU56	0	7	0	0	0	0	0	0	0	0	0	0
OTU57	0	0	0	0	0	0	0	0	350	0	0	0
OTU58	0	0	0	0	9	0	18	9	0	0	26	0
OTU59	0	0	0	0	0	2	0	0	0	1	0	0
OTU60	0	0	0	49	0	1	3	44	0	0	0	0
OTU61	0	0	0	1	0	0	0	10	0	0	0	0
OTU62	0	0	0	0	0	0	0	8	0	0	0	0
OTU63	0	0	0	0	0	0	11	0	0	0	0	0
OTU64	1	0	0	10	0	0	0	1	0	0	0	1
OTU65	0	810	0	0	0	0	0	0	0	0	2	0
OTU66	0	0	0	0	0	0	16	7	0	23	0	0
OTU67	4	1	0	9	0	0	0	0	0	0	0	0
OTU68	0	0	0	0	0	0	1	1	0	0	0	0
OTU69	0	0	0	0	0	0	0	11	0	0	0	0
OTU70	0	17	0	0	0	0	0	0	0	0	0	0
OTU71	0	0	0	0	0	5	0	0	0	0	0	0
OTU72	0	0	0	1	0	1	1	1	2	0	0	0
OTU73	0	0	0	0	0	1	44	0	1	0	0	0
OTU74	24	3	0	63	6	12	123	153	1	663	0	0
OTU75	6	0	0	0	0	0	0	0	0	0	0	0
OTU76	0	1032	0	1	0	0	0	39	0	0	4	0
OTU77	0	0	252	0	0	0	0	0	0	0	0	0
OTU78	0	0	0	0	0	0	0	76	0	0	0	1
OTU79	0	204	0	0	0	0	0	0	13	0	0	0
OTU80	1	3	0	0	0	0	0	0	65	156	0	0
OTU81	0	18	0	0	0	0	0	0	0	0	0	0
OTU82	0	0	0	37	952	2310	27	60	8	0	28	11
OTU83	5	0	42	0	0	0	0	0	0	0	0	0

OTU84	0	36	0	0	0	0	0	0	0	0	0	0
OTU85	0	6	0	0	0	0	0	264	49	1507	0	0
OTU86	0	0	0	33	0	4	0	260	0	0	0	0
OTU87	56	6	0	0	7	8	2	2	144	18	5	0
OTU88	0	0	0	0	0	0	0	17	0	6	0	0
OTU89	387	0	1	0	0	0	0	0	0	0	0	0
OTU90	0	15	0	0	0	0	0	0	0	0	0	0
OTU91	0	2	20	0	0	0	0	0	0	0	0	0
OTU92	0	0	0	11	2	0	0	2	0	0	0	0
OTU93	0	0	0	5	1190	172	0	16	0	0	0	0
OTU94	5	0	0	0	0	0	0	0	0	0	0	572
OTU95	2	0	0	84	30	11	2	1	0	0	0	1
OTU96	0	0	0	0	0	0	0	5	2	0	0	3
OTU97	0	160	0	0	0	0	0	0	0	0	0	0
OTU98	1	0	2	0	0	0	0	0	0	0	0	0
OTU99	0	0	0	4	0	0	0	0	1	0	0	0
OTU100	0	2	0	0	2	0	0	0	3	2	1	0
OTU101	0	0	0	0	8	0	0	0	0	0	0	0
OTU102	39	0	0	0	0	0	0	0	0	0	0	0
OTU103	0	0	0	47	0	0	23	267	0	0	0	0
OTU104	0	0	1	0	0	0	0	0	1	0	0	0
OTU105	0	0	0	1	0	0	0	9	2	391	0	0
OTU106	0	3	0	0	0	0	0	0	0	0	0	0
OTU107	0	0	0	0	0	0	0	0	0	14	0	0
OTU108	0	3	0	0	0	0	0	0	0	0	0	0
OTU109	0	0	0	0	0	0	1	0	3	0	0	0
OTU110	1	92	0	0	0	0	0	0	0	0	0	0
OTU111	0	495	1	0	120	436	3	3	2	0	0	0
OTU112	0	70	0	0	74	0	1	8	24	7	0	0
OTU113	0	0	0	0	0	5	0	0	0	0	0	0
OTU114	0	0	0	0	0	0	0	3	0	0	0	0
OTU115	5	0	0	0	0	0	0	0	0	0	0	0
OTU116	0	0	1	74	44	2	0	11	11	2	0	1
OTU117	0	9	0	0	0	0	0	0	0	0	0	0
OTU118	0	0	0	0	0	0	5	0	0	0	0	0
OTU119	1	0	230	0	0	0	0	0	0	0	0	0
OTU120	44	1	0	228	59	18	56	34	0	29	0	0
OTU121	4	0	0	0	0	0	0	0	0	0	0	0
OTU122	4	0	0	0	0	0	0	0	0	0	0	562
OTU123	0	0	1	0	0	138	0	1	0	2	0	1
OTU124	0	85	0	0	0	0	0	0	0	0	0	0
OTU125	1	0	0	117	13	3	1	2	0	0	0	24
OTU126	0	1	0	0	0	0	0	1	0	0	0	0

OTU127	1	10	0	0	0	0	0	0	0	0	0	0
OTU128	1	6	0	2	1	0	0	2	22	671	0	0
OTU129	0	0	0	1	18	8	0	0	0	0	0	0
OTU130	0	0	0	0	0	0	0	0	9	0	0	0
OTU131	2	0	274	0	0	0	0	0	0	0	0	0
OTU132	0	0	0	27	0	7	0	68	0	0	0	0
OTU133	0	44	0	0	0	0	0	0	0	0	0	0
OTU134	1164	0	48	0	0	0	1	0	0	0	0	0
OTU135	0	0	0	11	0	0	0	0	0	0	0	0
OTU136	0	0	0	0	0	0	0	6	0	0	0	0
OTU137	0	0	1	433	123	10	75	71	519	11	0	7
OTU138	0	0	0	14	0	3	0	122	0	0	0	0
OTU139	0	0	2	0	0	0	0	0	67	0	0	0
OTU140	0	7	0	0	0	0	0	0	0	0	0	0
OTU141	3	0	0	1	0	15	1	0	4	0	0	0
OTU142	0	1	0	0	0	0	1	18	0	0	579	37
OTU143	3	0	0	0	0	0	0	0	0	0	0	0
OTU144	0	24	0	0	0	0	0	0	0	0	0	0
OTU145	306	0	0	0	0	0	0	0	0	0	0	0
OTU146	0	1	0	0	0	0	2	5	0	1	0	0
OTU147	0	0	0	0	37	0	1	0	0	0	0	0
OTU148	0	4	0	0	0	0	0	0	1	0	0	0
OTU149	0	0	0	0	0	0	0	14	2	0	0	12
OTU150	0	0	0	0	0	1	19	0	0	0	0	0
OTU151	0	0	0	1	0	1	7	1	0	0	0	0
OTU152	0	0	0	30	0	0	0	0	0	0	0	0
OTU153	0	0	0	0	0	0	5	0	0	0	0	0
OTU154	0	0	0	0	12	0	0	0	0	0	0	0
OTU155	0	13	0	0	0	0	0	0	1	0	0	0
OTU156	0	0	0	7	0	0	0	0	0	0	0	0
OTU157	15	0	3	0	0	0	0	0	0	0	0	2365
OTU158	0	3	0	0	0	0	0	0	0	0	0	0
OTU159	0	0	0	9	339	27	1	13	82	0	0	0
OTU160	0	12	0	0	0	0	0	0	0	0	0	0
OTU161	0	0	0	0	0	0	0	1	0	24	0	0
OTU162	3	0	9	0	0	0	0	0	0	0	0	0
OTU163	0	114	0	0	0	0	0	1	0	0	0	0
OTU164	0	0	0	0	4	0	213	1	1	6	0	0
OTU165	0	19	0	0	0	0	0	0	0	0	0	0
OTU166	1	1	1	0	2	1679	0	0	0	3	0	9
OTU167	0	0	0	0	0	0	0	0	7	0	0	0
OTU168	0	0	0	14	16	86	7	35	1	0	30	0
OTU169	0	0	0	0	0	0	0	276	0	0	0	0

OTU170	0	9	0	0	40	0	0	0	0	4	0	0
OTU171	0	48	0	10	0	0	0	0	41	0	0	0
OTU172	0	0	0	0	0	0	0	0	0	0	587	0
OTU173	0	0	0	1	3	0	2	0	0	0	0	0
OTU174	0	0	0	6	52	14	11	9	297	0	2	0
OTU175	0	0	0	0	4	3	0	3	0	0	0	0
OTU176	1	0	343	0	0	0	0	0	3	0	0	0
OTU177	0	0	0	0	0	0	0	0	5	0	0	0
OTU178	0	23	0	0	0	0	0	0	0	0	0	0
OTU179	0	0	0	0	5	2	17	0	680	0	2	0
OTU180	0	0	0	0	0	0	0	3	3	215	0	0
OTU181	11	1	1	0	0	0	0	0	11	0	0	0
OTU182	0	0	0	0	10	3	12	2	1037	0	2	0
OTU183	1	0	0	0	0	0	0	0	0	0	0	495
OTU184	0	65	0	0	0	0	0	0	0	0	0	0
OTU185	0	6	0	0	0	0	0	104	3	1083	0	0
OTU186	3	0	2	2010	152	45	9	31	0	5	0	137
OTU187	92	213	8	0	0	0	0	0	0	0	1	0
OTU188	0	0	0	0	0	0	2	0	0	0	0	0
OTU189	0	0	2	297	0	49	0	3	0	1	0	0
OTU190	0	0	0	0	0	0	0	0	10	0	0	0
OTU191	49	0	0	5	419	43	249	0	0	0	0	2
OTU192	0	0	0	0	0	0	8	0	0	0	0	0
OTU193	0	0	0	0	0	0	0	0	101	0	0	0
OTU194	0	1	0	0	47	0	0	0	0	0	0	0
OTU195	0	0	0	0	8	0	0	0	2	0	0	0
OTU196	0	0	0	0	22	0	15	0	0	0	0	0
OTU197	0	0	0	0	0	0	123	0	2	0	0	0
OTU198	0	0	0	0	0	0	4	0	4	0	0	0
OTU199	0	17	0	0	0	0	0	0	0	0	0	0
OTU200	41	0	1	0	0	0	0	0	0	0	0	0
OTU201	317	0	16	0	0	0	0	0	0	0	0	0
OTU202	3	0	0	0	0	0	0	0	11	2	0	0
OTU203	0	0	0	1	4	0	90	83	20	0	3	0
OTU204	9	4	1	0	0	0	0	0	0	0	0	0
OTU205	0	0	0	0	0	1	1	3	1	0	0	0
OTU206	1	0	0	0	1	0	24	34	0	0	1695	89
OTU207	0	0	0	0	1	3242	0	0	0	3	0	6
OTU208	4	16	0	0	0	0	0	0	0	0	0	0
OTU209	0	156	0	0	0	0	0	0	0	0	0	0
OTU210	0	0	0	0	0	0	1	0	0	78	0	0
OTU211	0	3	0	0	0	0	0	0	0	0	0	0
OTU212	1	0	11	9	1	0	0	4	5	6	0	0

OTU213	0	0	0	1	19	0	66	0	13	0	0	0
OTU214	0	0	0	0	12	0	0	0	0	0	0	0
OTU215	2	0	0	1151	66	19	23	15	4	1	2	197
OTU216	0	0	0	0	0	0	0	12	0	0	0	0
OTU217	0	0	0	0	2	0	0	0	0	0	0	0
OTU218	0	0	0	0	0	0	0	0	164	0	0	0
OTU219	0	7	0	2	0	0	0	0	3	0	0	0
OTU220	0	0	0	0	25	1	0	3	0	0	0	0
OTU221	180	15	615	0	0	0	0	0	0	0	0	0
OTU222	1	0	0	180	9	6	23	3	2	0	0	42
OTU223	2650	9	2085	1	0	0	2	0	2	0	0	0
OTU224	0	0	27	0	0	0	0	0	0	0	0	0
OTU225	0	14	0	0	0	0	0	0	0	0	0	0
OTU226	0	0	0	0	0	0	0	0	3	0	0	0
OTU227	6	5	0	25	8	0	0	0	0	0	0	0
OTU228	3	0	0	461	146	133	32	0	0	0	0	1
OTU229	0	0	0	0	0	0	61	0	0	0	1	0
OTU230	11	12	0	15	4	0	0	0	1	0	2	0
OTU231	0	7	0	0	1	0	27	99	0	101	0	0
OTU232	0	0	0	1	36	1	271	6	2	13	9	0
OTU233	10	0	80	0	0	0	0	0	1	0	0	0
OTU234	0	0	0	0	0	1	44	0	0	0	1	0
OTU235	0	0	0	0	0	0	0	0	153	0	0	0
OTU236	0	0	0	0	0	0	0	9	0	6	0	0
OTU237	1	1	0	0	0	0	0	0	0	0	406	0
OTU238	0	0	0	11	0	0	0	7	0	0	0	0
OTU239	0	47	0	0	0	0	0	0	0	0	1	0
OTU240	0	0	0	0	0	118	37	0	0	0	0	0
OTU241	0	0	0	0	0	7	3	0	1	0	0	0
OTU242	0	0	0	0	0	0	0	5	0	0	0	0
OTU243	0	0	0	0	0	0	38	0	0	0	0	0
OTU244	0	0	0	0	0	0	0	0	1042	3	0	0
OTU245	0	123	0	0	112	0	9	17	501	17	0	0
OTU246	2	1	0	0	0	0	0	0	0	0	0	0
OTU247	0	0	0	594	1	80	0	3101	0	0	0	0
OTU248	0	22	0	0	0	0	0	0	0	0	0	0
OTU249	1	0	0	0	0	180	497	0	0	0	0	0
OTU250	0	0	0	0	0	0	0	6	0	0	0	0
